# Low expression of TOX predicts poor prognosis of patients with breast cancer in the real world: A retrospective study

**DOI:** 10.1016/j.heliyon.2024.e41180

**Published:** 2024-12-12

**Authors:** Chunlei Tan, Danping Wu, Xiaotian Yang, Shiyuan Zhang, Shuqiang Liu, Boqian Yu, Xiao Yu, Yuting Xiu, Yuanxi Huang

**Affiliations:** Department of Breast Surgery, Harbin Medical University Cancer Hospital, Harbin, Heilongjiang, 150081, PR China

**Keywords:** Breast cancer, TOX, Chemotherapy, Prognosis, Survival

## Abstract

**Background:**

TOX is a transcription factor that is implicated in the regulation of T cell exhaustion in tumors. TOX has been proven to have prognostic value in some malignant tumors. We aim to analyze the expression of TOX in breast cancer patients, and the association between TOX and prognostic significance in patients with breast cancer.

**Methods:**

313 breast cancer patients were enrolled into this study. The expression of TOX was determined by immunohistochemistry assay. Survival curves were performed by Kaplan-Meier and log-rank test. The potential independent factors were assessed by Cox regression analyses. Nomogram models, calibration curve, decision curve analyses were applied to analyze the clinical utility of predictive models.

**Results:**

According to semi-quantitative scoring, 129 patients were classified into low group, and 184 patients were classified into high group. Patients with high expression of TOX had a longer survival than those with low expression of TOX (DFS: 71.70 vs. 64.05 months, χ^2^ = 11.6300, P = 0.00065; OS: 81.03 vs. 73.72 months, χ^2^ = 11.4200, P = 0.00073). Based on Cox regression analyses, multivariate analysis indicated that TOX was the potential prognostic factor for both DFS (HR: 0.412, 95 % CI: 0.248–0.684, P = 0.001) and OS (HR: 0.395, 95 % CI: 0.237–0.660, P < 0.0001). Calibration curve analysis showed that the predicted line was well-matched with baseline regarding postoperative 1-, 3-, and 5-year survival rate.

**Conclusions:**

The expression of TOX is a potential prognostic factor, and can be a promising biomarker for predicting survival in breast cancer patients.

## Highlights

• Discovery of varying TOX expression in breast cancer tissues offers novel insights.
• Revelation of low TOX expression as a predictor of poorer clinical outcomes.
• High expression of TOX correlates with longer survival, notably in stage II patients and those undergoing chemotherapy.
• Multivariate analysis reveals TOX as a prognostic indicator and therapeutic target.

## 1 Introduction

Breast cancer is one of the most common malignant tumors among women and a major threat to people's health in the world [1]. It is expected that there will be 2.26 million newly diagnosed cases and 680,000 cancer deaths among females worldwide in 2020 [2]. In clinical practice, immunohistochemical analysis of estrogen receptors (ER), progesterone receptors (PR), human epidermal growth factor receptor 2 (HER2), and Ki-67 has been conducted as alternative markers for determining breast cancer molecular subtypes [3]. According to gene expression profiles, breast cancer is usually divided into luminal A-like (ER+/HER2-), luminal B-like (ER+/HER2+ or ER+/HER2-), HER2-enriched (non-luminal, ER-/HER2+), and basal (triple-negative breast cancer, ER-/PR-/HER2-) [4]. Despite remarkable improvements having been made in surgical management and systematic treatment, high recurrence and metastasis rates, as well as chemotherapy resistance rates, are the main reasons for low survival rates [5,6]. For breast cancer, the tumor microenvironment including lymphoid and myeloid white blood cells plays important roles in disease progression and affects clinical outcomes [7]. Moreover, the immune cell composition of breast cancer varies depending on the molecular characteristics of the tumor [8].

Some studies have analyzed the sophisticated relationship between molecular characteristics of tumors and immune cells in the tumor microenvironment[[9], [10], [11]]. Immune checkpoint inhibitors (ICIs) are applied to treat various types of malignant tumors, including breast cancer [12,13]. However, the response of patients with different tumors to immune checkpoint inhibitors shows extensive therapeutic differences, with only some patients showing significant benefits [14,15]. Based on the clinical practice, the immune checkpoint proteins (ICPs) include PD-1, PD-L1, and CTLA-4 [16]. The interaction between PD-1 and its ligand PD-L1 on tumor cells and antigen-presenting cells affects T cells, leading to T cell exhaustion and dysfunction [17]. Consequently, the identification of effective biomarkers can prognosticate the response and clinical outcomes to enhance cancer immunotherapy, and may play a notable role in the treatment of this malignant tumor.

TOX belongs to a large superfamily of high mobility group (HMG) box proteins and nuclear DNA-binding factor, and includes a small family of four proteins [18]. TOX is a key transcription factor related to the development of malignant tumors and plays a significant role in T cells and other lymphocytes [19]. As a key regulatory factor for T cell differentiation, TOX has been found to be involved in CD8+T cell exhaustion and is driven by epigenetic reprogramming of CD8+T cells [20,21]. Thus, TOX may influence the function of CD8^+^ T cell, and predict prognosis for cancer patients. Several studies have demonstrated that TOX was related to tumor size, TNM stage, differentiation, and acted as a potential biomarker for cancer treatment[[22], [23], [24]]. However, the correlation among TOX and prognosis of breast cancer is yet not clear.

In this research, we comprehensively investigated the relationship between the expression level of TOX and clinical characteristics in breast cancer patients. The Cox model regression analyses were applied to distinguish the potential prognostic factors. We also explored its relationship with the survival of breast cancer patients. Thus, this study discovered that TOX was a potential prognostic biomarker, and high expression of TOX predicted good prognosis of patients with breast cancer, which provided a new direction for predicting survival of breast cancer patients.

## 2 Materials and methods

### 2.1 Patients and specimens

For this study, the formalin-fixed paraffin-embedded (FFPE) tissues were gleaning from 313 breast cancer patients from January 2015 to November 2015. This study was approved by the Ethics Committee of Harbin Medical University Cancer Hospital with the approval number KY2023-38. All patients provided written informed consent to participate in the study and for their data to be published. Inclusion criteria were as follows: 1) diagnosed with breast cancer; 2) received curative surgery for all selected patients; 3) complete follow-up information. Exclusion criteria were as follows: 1) with metastasis or other malignant tumors; 2) received anti-tumor treatment before surgery in our hospital; 3) accompanied by acute or chronic inflammatory diseases, and were difficult to control.

### 2.2 Immunohistochemistry staining assay

Immunohistochemistry staining assay was followed by the standard protocols: (1) Paraffin-embedded the breast cancer patients’ tissues. (2) Paraffin slicing and dewaxing: Place the slices sequentially in xylene I and II for 15min, anhydrous ethanol I and II for 5min, 85 % and 75 % alcohol for 5min, followed by washing with distilled water. (3) Antigen retrieval with EDTA antigen repair buffer (pH 9.0). (4) Blocking endogenous peroxidase. (5) Serum blockade: added 3 % BSA into the tissue for 30 min (6) Added primary antibody: TOX (1:200 dilution, Ab155768, Abcam, Shanghai, China) was added and incubated TOX antibody overnight in a wet box at 4 °C. (7) Added secondary antibody: the goat anti-rabbit IgG H&L (1:200 dilution, GB23303, Servicebio, Wuhan, China) was added and incubated for 1 h in a wet box at room temperature. (8) DAB color rendering. (9) Recombinant staining of cell nucleus. (10) Dehydration and sealing. (11) Microscopic examination.

### 2.3 Evaluation methods for clinical pathological parameters

The Union for International Cancer Control (UICC) and American Joint Committee on Cancer staging system (AJCC) were used for cancer staging. The expression of TOX was obtained by immunohistochemistry, and evaluated based on the density and intensity of stained cells. The density of positively stained cells was as follows: (1) 0-score: less than 1 % stained; (2) 1-score: 1%–10 %; (3) 2-score: 11%–50 %; (4) 3-score: 51%–75 %; (5) 4-score: 76%–100 %. The intensity of positively stained cells was as follows: (1) 0-score: no staining; (2) 1-score: light yellow staining; (3) 2-score: brown yellow dyeing; (4) 3-score: yellowish brown dyeing. In the present study, patients were divided into two groups: low TOX expression (the score was under 4 scores) and high TOX expression (the score was more than 4 scores).

### 2.4 Followed-up

In this study, all patients were followed by inpatient, outpatient, or telephone calls after surgery. Follow up plan for postoperative patients as follows: 1) every three months in the first year after surgery, 2) every six months in the second year and third year after surgery; 3) once a year until death. Disease free survival (DFS) referred to the time from surgical resection until any local or remote metastasis of breast cancer or death from any cause. Overall survival (OS) was evaluated from the time after curative resection to death due to any reason or last follow-up.

### 2.5 Statistical analysis

SPSS Statistics software 22.0 (IBM Corp.) and R (version 4.2.2; Vienna, Austria. URL: https://www.r-project.org/) were used to perform all statistical analyses. The associations between TOX and clinicopathological variables in breast cancer were performed by chi-square test. Survival curves were estimated by Kaplan-Meier method and log-rank test. The potential independent factors were assessed by the univariate and multivariate Cox model analyses. Nomogram models were established to evaluate the DFS and OS rate. The calibration curve and decision curve analyses were applied to analyze clinical utility of predictive models.

## 3 Results

### 3.1 The expression of TOX in breast cancer tissues

**Fig. 1:**
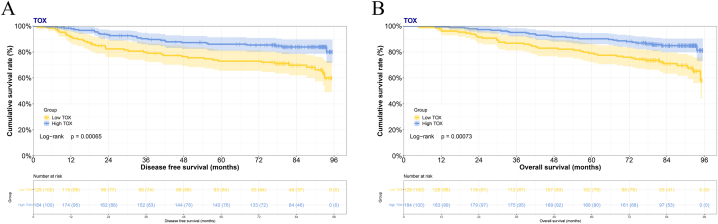
Survival curve of TOX expression level and prognosis in patients with breast cancer for (A) disease free survival and (B) overall survival.

The expression of TOX in surgical specimens of breast cancer were detected by immunohistochemistry staining. According the semi-quantitative scoring under electron microscope, 129 breast cancer patient samples were observed to under 4 scores, and 184 breast cancer patient samples were observed to more than 4 scores. Fig. S1 shown the representative figures for the expression of TOX. The average expression of TOX in breast cancer tissues was much higher than in the adjacent normal tissue to breast cancer. Of these breast cancer patients, patients with high level expression of TOX (184 cases) had longer survival time than those with low level expression of TOX (129 cases) (DFS: 71.70 vs. 64.05 months; OS: 81.03 vs. 73.72 months), and the difference in survival time between the two groups was statistically significant (DFS: χ^2^ = 11.6300, P = 0.00065; OS: χ^2^ = 11.4200, P = 0.00073) (Fig. 1A and B). We also analyzed the TOX expression in Kaplan-Meier Plotter database, the results also shown that patients with high level expression of TOX (RFS 2463 cases, OS 934 cases) had longer survival time than those with low level expression of TOX (RFS 2466 cases, OS 945 cases), and the difference in survival time between the two groups was statistically significant (RFS: P < 0.00001; OS:P = 0.00022) (Figs. S2A and B).

### 3.2 Demographic features

Based on the TOX expression, these patients were divided into two groups: 129 cases in low group, and 184 cases in high group. The median age was 51 years (range from 25 to 78 years). The median menarche age was 15 years, and ranged from 12 to 23 years. Compared with these patients, the TOX expression level was dramatically concerned with operative time (P = 0.03). The detail information was shown in Table 1.

**Table 1:** Demographic and clinicopathologic characteristics of patients with breast cancer.

	level	Overall	Low TOX	High TOX	p
n		313	129	184	
Age	<51	154 (49.2)	68 (52.7)	86 (46.7)	0.355
	≥51	159 (50.8)	61 (47.3)	98 (53.3)	
BMI	<23.8	156 (49.8)	67 (51.9)	89 (48.4)	0.612
	≥23.8	157 (50.2)	62 (48.1)	95 (51.6)	
Family history	No	242 (77.3)	97 (75.2)	145 (78.8)	0.539
	Yes	71 (22.7)	32 (24.8)	39 (21.2)	
Basic disease	No	244 (78.0)	97 (75.2)	147 (79.9)	0.396
	Yes	69 (22.0)	32 (24.8)	37 (20.1)	
Hypertension	No	273 (87.2)	109 (84.5)	164 (89.1)	0.300
	Yes	40 (12.8)	20 (15.5)	20 (10.9)	
Diabetes mellitus	No	297 (94.9)	123 (95.3)	174 (94.6)	0.961
	Yes	16 (5.1)	6 (4.7)	10 (5.4)	
Coronary heart disease	No	300 (95.8)	123 (95.3)	177 (96.2)	0.935
	Yes	13 (4.2)	6 (4.7)	7 (3.8)	
Menarche age	<15	121 (38.7)	50 (38.8)	71 (38.6)	1.000
	≥15	192 (61.3)	79 (61.2)	113 (61.4)	
Menopause	No	152 (48.6)	65 (50.4)	87 (47.3)	0.670
	Yes	161 (51.4)	64 (49.6)	97 (52.7)	
Blood type	A	72 (23.0)	25 (19.4)	47 (25.5)	0.350
	B	118 (37.7)	46 (35.7)	72 (39.1)	
	O	89 (28.4)	42 (32.6)	47 (25.5)	
	AB	34 (10.9)	16 (12.4)	18 (9.8)	
Primary tumor site	Upper outer quadrant	178 (56.9)	72 (55.8)	106 (57.6)	0.403
	Lower outer quadrant	29 (9.3)	9 (7.0)	20 (10.9)	
	Lower inner quadrant	23 (7.3)	13 (10.1)	10 (5.4)	
	Upper inner quadrant	44 (14.1)	17 (13.2)	27 (14.7)	
	Central	39 (12.5)	18 (14.0)	21 (11.4)	
US-BIRADS	BIRADS 4	173 (55.3)	70 (54.3)	103 (56.0)	0.930
	BIRADS 5	129 (41.2)	54 (41.9)	75 (40.8)	
	BIRADS 6	11 (3.5)	5 (3.9)	6 (3.3)	
Operative time (min)	<75	143 (45.7)	49 (38.0)	94 (51.1)	0.030
	≥75	170 (54.3)	80 (62.0)	90 (48.9)	
Type of surgery	Mastectomy	293 (93.6)	117 (90.7)	176 (95.7)	0.126
	Breast-conserving surgery	20 (6.4)	12 (9.3)	8 (4.3)	
Tumor size (cm)	≤2	154 (49.2)	69 (53.5)	85 (46.2)	0.441
	>2 and < 5	149 (47.6)	56 (43.4)	93 (50.5)	
	≥5	10 (3.2)	4 (3.1)	6 (3.3)	
Histologic grade	I	8 (2.6)	4 (3.1)	4 (2.2)	0.510
	II	183 (58.5)	79 (61.2)	104 (56.5)	
	III	106 (33.9)	38 (29.5)	68 (37.0)	
	Unknown	16 (5.1)	8 (6.2)	8 (4.3)	
Pathological T Stage	T1	167 (53.4)	72 (55.8)	95 (51.6)	0.887
	T2	134 (42.8)	52 (40.3)	82 (44.6)	
	T3	10 (3.2)	4 (3.1)	6 (3.3)	
	T4	2 (0.6)	1 (0.8)	1 (0.5)	
Pathological N Stage	N0	129 (41.2)	48 (37.2)	81 (44.0)	0.204
	N1	97 (31.0)	39 (30.2)	58 (31.5)	
	N2	50 (16.0)	21 (16.3)	29 (15.8)	
	N3	37 (11.8)	21 (16.3)	16 (8.7)	
Pathological TNM Stage	I	85 (27.2)	36 (27.9)	49 (26.6)	0.217
	II	138 (44.1)	50 (38.8)	88 (47.8)	
	III	90 (28.8)	43 (33.3)	47 (25.5)	
TLN	<16	149 (47.6)	68 (52.7)	81 (44.0)	0.161
	≥16	164 (52.4)	61 (47.3)	103 (56.0)	
PLN	<1	134 (42.8)	50 (38.8)	84 (45.7)	0.273
	≥1	179 (57.2)	79 (61.2)	100 (54.3)	
TALN	<14	149 (47.6)	70 (54.3)	79 (42.9)	0.063
	≥14	164 (52.4)	59 (45.7)	105 (57.1)	
PALN	<1	157 (50.2)	60 (46.5)	97 (52.7)	0.334
	≥1	156 (49.8)	69 (53.5)	87 (47.3)	
Molecular subtype	Luminal A	58 (18.5)	27 (20.9)	31 (16.8)	0.916
	Luminal B HER2+	63 (20.1)	25 (19.4)	38 (20.7)	
	Luminal B HER2-	63 (20.1)	26 (20.2)	37 (20.1)	
	HER2 enriched	65 (20.8)	25 (19.4)	40 (21.7)	
	Triple negative	64 (20.4)	26 (20.2)	38 (20.7)	
ER	0–25 %	144 (46.0)	61 (47.3)	83 (45.1)	0.961
	26–50 %	26 (8.3)	11 (8.5)	15 (8.2)	
	51–75 %	48 (15.3)	20 (15.5)	28 (15.2)	
	76–100 %	95 (30.4)	37 (28.7)	58 (31.5)	
PR	0–25 %	192 (61.3)	81 (62.8)	111 (60.3)	0.433
	26–50 %	35 (11.2)	10 (7.8)	25 (13.6)	
	51–75 %	35 (11.2)	15 (11.6)	20 (10.9)	
	76–100 %	51 (16.3)	23 (17.8)	28 (15.2)	
HER2	Negative	185 (59.1)	79 (61.2)	106 (57.6)	0.599
	Positive	128 (40.9)	50 (38.8)	78 (42.4)	
Ki67	0–25 %	147 (47.0)	70 (54.3)	77 (41.8)	0.143
	26–50 %	105 (33.5)	39 (30.2)	66 (35.9)	
	51–75 %	46 (14.7)	14 (10.9)	32 (17.4)	
	76–100 %	15 (4.8)	6 (4.7)	9 (4.9)	
CK5/6	Negative	221 (70.6)	93 (72.1)	128 (69.6)	0.721
	Positive	92 (29.4)	36 (27.9)	56 (30.4)	
E-cad	Negative	11 (3.5)	8 (6.2)	3 (1.6)	0.064
	Positive	302 (96.5)	121 (93.8)	181 (98.4)	
P120	Negative	293 (93.6)	118 (91.5)	175 (95.1)	0.289
	Positive	20 (6.4)	11 (8.5)	9 (4.9)	
P53	Negative	170 (54.3)	78 (60.5)	92 (50.0)	0.086
	Positive	143 (45.7)	51 (39.5)	92 (50.0)	
Blood vessel invasion	No	289 (92.3)	120 (93.0)	169 (91.8)	0.866
	Yes	24 (7.7)	9 (7.0)	15 (8.2)	
Chemotherapy	No	23 (7.3)	11 (8.5)	12 (6.5)	0.653
	Yes	290 (92.7)	118 (91.5)	172 (93.5)	
Radiotherapy	No	220 (70.3)	86 (66.7)	134 (72.8)	0.295
	Yes	93 (29.7)	43 (33.3)	50 (27.2)	
Endocrine therapy	No	150 (47.9)	59 (45.7)	91 (49.5)	0.594
	Yes	163 (52.1)	70 (54.3)	93 (50.5)	
Targeted therapy	No	279 (89.1)	113 (87.6)	166 (90.2)	0.583
	Yes	34 (10.9)	16 (12.4)	18 (9.8)	

### 3.3 Associations between TOX expression and metastasis data in breast cancer

In this study, of these patients, some patients developed liver metastasis (29 cases), lung metastasis (32 cases), brain metastasis (16 cases), bone metastasis (40 cases), and so forth. The detail information was shown in Table 2. The expression of TOX was concerned with liver metastasis (P < 0.05).

**Table 2:** Associations between TOX expression and metastasis in breast cancer.

	level	Overall	Low TOX	High TOX	p
n		313	129	184	
Lung metastasis	No	281 (89.8)	115 (89.1)	166 (90.2)	0.906
	Yes	32 (10.2)	14 (10.9)	18 (9.8)	
Bone metastasis	No	273 (87.2)	110 (85.3)	163 (88.6)	0.488
	Yes	40 (12.8)	19 (14.7)	21 (11.4)	
Liver metastasis	No	284 (90.7)	111 (86.0)	173 (94.0)	0.028
	Yes	29 (9.3)	18 (14.0)	11 (6.0)	
Mediastinal metastasis	No	299 (95.5)	121 (93.8)	178 (96.7)	0.337
	Yes	14 (4.5)	8 (6.2)	6 (3.3)	
Brain metastasis	No	297 (94.9)	119 (92.2)	178 (96.7)	0.130
	Yes	16 (5.1)	10 (7.8)	6 (3.3)	
Pleural metastasis	No	302 (96.5)	124 (96.1)	178 (96.7)	1.000
	Yes	11 (3.5)	5 (3.9)	6 (3.3)	
Chest wall metastasis	No	301 (96.2)	125 (96.9)	176 (95.7)	0.790
	Yes	12 (3.8)	4 (3.1)	8 (4.3)	
Axillary metastasis	No	154 (49.2)	60 (46.5)	94 (51.1)	0.495
	Yes	159 (50.8)	69 (53.5)	90 (48.9)	
Clavicle metastasis	No	264 (84.3)	110 (85.3)	154 (83.7)	0.826
	Yes	49 (15.7)	19 (14.7)	30 (16.3)	

### 3.4 Relationship between TOX expression and common hematological parameters

In the current study, these common hematological parameters were grouped into two by the median values of these parameters. Compared with the two groups by TOX expression, significant associations were found for CA153 (P = 0.043), and D-D (P = 0.033). The detail information was shown in Table 3.

**Table 3:** Relationship between TOX expression and common hematological parameters.

	level	Overall	Low TOX	High TOX	p
n		313	129	184	
ALT	<21	142 (45.4)	55 (42.6)	87 (47.3)	0.485
	≥21	171 (54.6)	74 (57.4)	97 (52.7)	
AST	<23	147 (47.0)	53 (41.1)	94 (51.1)	0.103
	≥23	166 (53.0)	76 (58.9)	90 (48.9)	
AST/ALT	<1.1	155 (49.5)	66 (51.2)	89 (48.4)	0.710
	≥1.1	158 (50.5)	63 (48.8)	95 (51.6)	
LDH	<170	156 (49.8)	64 (49.6)	92 (50.0)	1.000
	≥170	157 (50.2)	65 (50.4)	92 (50.0)	
GGT	<14	142 (45.4)	56 (43.4)	86 (46.7)	0.641
	≥14	171 (54.6)	73 (56.6)	98 (53.3)	
ALP	<70	154 (49.2)	64 (49.6)	90 (48.9)	0.994
	≥70	159 (50.8)	65 (50.4)	94 (51.1)	
GLU	<5.1	147 (47.0)	63 (48.8)	84 (45.7)	0.659
	≥5.1	166 (53.0)	66 (51.2)	100 (54.3)	
ALB	<45	145 (46.3)	58 (45.0)	87 (47.3)	0.772
	≥45	168 (53.7)	71 (55.0)	97 (52.7)	
BUN	<4.9	151 (48.2)	67 (51.9)	84 (45.7)	0.327
	≥4.9	162 (51.8)	62 (48.1)	100 (54.3)	
UR/CR	<0.078	154 (49.2)	66 (51.2)	88 (47.8)	0.641
	≥0.078	159 (50.8)	63 (48.8)	96 (52.2)	
CRE	<63	150 (47.9)	63 (48.8)	87 (47.3)	0.876
	≥63	163 (52.1)	66 (51.2)	97 (52.7)	
URIC	<253	156 (49.8)	63 (48.8)	93 (50.5)	0.855
	≥253	157 (50.2)	66 (51.2)	91 (49.5)	
TBIL	<12.45	156 (49.8)	57 (44.2)	99 (53.8)	0.119
	≥12.45	157 (50.2)	72 (55.8)	85 (46.2)	
DBIL	<3.9	155 (49.5)	56 (43.4)	99 (53.8)	0.090
	≥3.9	158 (50.5)	73 (56.6)	85 (46.2)	
IBIL	<8.29	156 (49.8)	57 (44.2)	99 (53.8)	0.119
	≥8.29	157 (50.2)	72 (55.8)	85 (46.2)	
TP	<74	132 (42.2)	54 (41.9)	78 (42.4)	1.000
	≥74	181 (57.8)	75 (58.1)	106 (57.6)	
G	<29	137 (43.8)	57 (44.2)	80 (43.5)	0.993
	≥29	176 (56.2)	72 (55.8)	104 (56.5)	
A/G	<1.5	104 (33.2)	36 (27.9)	68 (37.0)	0.121
	≥1.5	209 (66.8)	93 (72.1)	116 (63.0)	
PAB	<267	156 (49.8)	59 (45.7)	97 (52.7)	0.271
	≥267	157 (50.2)	70 (54.3)	87 (47.3)	
CO2	<28.5	156 (49.8)	62 (48.1)	94 (51.1)	0.680
	≥28.5	157 (50.2)	67 (51.9)	90 (48.9)	
CA153	<9.82	156 (49.8)	55 (42.6)	101 (54.9)	0.043
	≥9.82	157 (50.2)	74 (57.4)	83 (45.1)	
CEA	<1.49	156 (49.8)	65 (50.4)	91 (49.5)	0.962
	≥1.49	157 (50.2)	64 (49.6)	93 (50.5)	
D-D	<0.25	151 (48.2)	72 (55.8)	79 (42.9)	0.033
	≥0.25	162 (51.8)	57 (44.2)	105 (57.1)	
FBG	<2.6	153 (48.9)	69 (53.5)	84 (45.7)	0.211
	≥2.6	160 (51.1)	60 (46.5)	100 (54.3)	
INR	<0.97	139 (44.4)	58 (45.0)	81 (44.0)	0.961
	≥0.97	174 (55.6)	71 (55.0)	103 (56.0)	
PT	<11.1	138 (44.1)	58 (45.0)	80 (43.5)	0.885
	≥11.1	175 (55.9)	71 (55.0)	104 (56.5)	
APTT	<27.5	152 (48.6)	64 (49.6)	88 (47.8)	0.844
	≥27.5	161 (51.4)	65 (50.4)	96 (52.2)	
TT	<17.2	154 (49.2)	58 (45.0)	96 (52.2)	0.254
	≥17.2	159 (50.8)	71 (55.0)	88 (47.8)	

### 3.5 Subgroup analysis for adjuvant therapy by TOX expression after surgery in breast cancer

Of these patients, there were 290 cases received chemotherapy. Based on the TOX expression, these patients were divided into two groups: 118 cases in low group and 172 cases in high group. Patients with high expression of TOX (172 cases) had longer survival time than those patients with low expression of TOX (118 cases) (DFS: 72.85 vs. 64.53 months, P = 0.00029; OS: 82.06 vs. 74.29 months, P = 0.00033) (Fig. S3). For these received chemotherapy patients, the clinical and pathological features were shown in Table S1. The expression of TOX was related to liver metastasis (P = 0.013). The common toxic side effects of chemotherapy were mainly gastrointestinal reactions and myelosuppression. There was no difference between the two groups among these common toxic side effects.

Of these patients, there were 163 cases received endocrine therapy. Based on the TOX expression, these patients were divided into two groups: 70 cases in low group and 93 cases in high group. Patients with high expression of TOX (93 cases) had longer survival time than those patients with low expression of TOX (70 cases) (DFS: 87.77 vs. 76.37 months, P = 0.41; OS: 88.77 vs. 83.87 months, P = 0.49) (Fig. S4). For these received endocrine therapy patients, the clinical and pathological features were shown in Table S2. The expression of TOX was related to primary tumor site (P = 0.020), operative time (P = 0.029), P53 (P = 0.010), liver metastasis (P = 0.049), endocrine therapy drugs (P = 0.017).

Of these patients, there were 93 cases received radiotherapy. Based on the TOX expression, these patients were divided into two groups: 43 cases in low group and 50 cases in high group. Patients with high expression of TOX (50 cases) had longer survival time than those patients with low expression of TOX (DFS: 64.56 vs. 61.78 months, P = 0.48; OS: 81.24 vs. 76.52 months, P = 0.39) (43 cases) (Fig. S5). For these received radiotherapy patients, the clinical and pathological features were shown in Table S3. The expression of TOX was related to D-D (P = 0.047).

### 3.6 Subgroup analysis for TNM stage and molecular subtype by TOX expression after surgery in breast cancer

In this study, 85 patients were stage I, 138 patients were stage II, and 90 patients were stage III. For stage I patients, these patients were divided into two groups: 36 cases in low group and 49 cases in high group by the TOX expression. Patients with high expression of TOX (49 cases) had longer survival time than those patients with low expression of TOX (36 cases) (DFS: 76.31 vs. 73.85 months, P = 0.23; OS: 81.30 vs. 80.22 months, P = 0.23) (Fig. S6). For this stage I patients, the clinical and pathological features were shown in Table S4. The expression of TOX was related to BUN (P = 0.010), CA153 (P = 0.042). For stage II patients, these patients were divided into two groups: 50 cases in low group and 88 cases in high group by the TOX expression. Patients with high expression of TOX (88 cases) had longer survival time than those patients with low expression of TOX (50 cases) (DFS: 75.10 vs. 72.34 months, P = 0.0140; OS: 84.45 vs. 78.87 months, OS: P = 0.0085) (Fig. S7). For these stage II patients, the clinical and pathological features were shown in Table S5. The expression of TOX was related to menopause (P = 0.041), TLN (P = 0.018), TALN (P = 0.004), P53 (P = 0.013). For stage III patients, these patients were divided into two groups: 43 cases in low group and 47 cases in high group by the TOX expression. Patients with higher expression of TOX (47 cases) had longer survival time than those patients with lower expression of TOX (43 cases) (DFS: 60.53 vs. 46.22 months, P = 0.071; OS: 74.33 vs. 62.30 months, P = 0.085) (Fig. S8). For these stage III patients, the clinical and pathological features were shown in Table S6. There was no difference between the two groups among these stage III patients.

According to the molecular subtype, 58 (18.5 %) cases were Luminal A subtype, 63 (20.1 %) cases were Luminal B HER2+ subtype, 63 (20.1 %) cases were Luminal B HER2-subtype, 65 (20.8 %) cases were HER2 enriched subtype, 64 (20.4 %) cases were triple negative subtype, respectively. In Luminal A subtype, there were 27 cases in the low TOX expression group and 31 cases in the high TOX expression group (DFS, χ^2^ = 2.5933, P = 0.1073; OS, χ^2^ = 2.7016, P = 0.1003). In Luminal B HER2+ subtype, there were 25 cases in the low TOX expression group and 38 cases in the high TOX expression group (DFS, χ^2^ = 1.9490, P = 0.1627; OS, χ^2^ = 1.8542, P = 0.1733). In Luminal B HER2-subtype, there were 26 cases in the low TOX expression group and 37 cases in the high TOX expression group (DFS, χ^2^ = 3.6149, P = 0.0573; OS, χ^2^ = 3.4432, P = 0.0635). In HER2 enriched subtype, there were 25 cases in the low TOX expression group and 40 cases in the high TOX expression group (DFS, χ^2^ = 5.9418, P = 0.0148; OS, χ^2^ = 7.0405, P = 0.0080). In Triple negative subtype, there were 26 cases in the low TOX expression group and 38 cases in the high TOX expression group (DFS, χ^2^ = 8.3918, P = 0.0038; OS, χ^2^ = 7.2460, P = 0.0071).

### 3.7 Cox proportional hazards model for univariate and multivariate analysis of the potential prognostic factors

Based on the Cox proportional hazards model for DFS, the multivariate analysis performed that TOX [hazard ratio (HR): 0.412, 95%CI: 0.248–0.684, P = 0.001)], pathological TNM Stage (HR: 6.305, 95%CI: 3.053–13.020, P < 0.0001), lung metastasis (HR: 2.855, 95%CI: 1.279–6.374, P = 0.010), bone metastasis (HR: 4.314, 95%CI: 2.022–9.204, P < 0.0001), brain metastasis (HR: 6.810, 95%CI: 3.150–14.723, P < 0.0001), chemotherapy (HR: 0.163, 95%CI: 0.079–0.337, P < 0.0001), radiotherapy (HR: 0.324, 95%CI: 0.157–0.668, P = 0.002), endocrine therapy (HR: 0.138, 95%CI: 0.073–0.260, P < 0.0001) were the potential prognostic factors (Table 4). According to the proportional hazards model for OS, the multivariate analysis indicated that TOX (HR: 0.395, 95%CI: 0.237–0.660, P < 0.0001), pathological TNM Stage (HR: 8.578, 95%CI: 3.973–18.524, P < 0.0001), lung metastasis (HR: 2.425, 95%CI: 1.279–4.597, P = 0.007), liver metastasis (HR: 3.558, 95%CI: 1.905–6.646, P < 0.0001), chemotherapy (HR: 0.139, 95%CI: 0.066–0.290, P < 0.0001), radiotherapy (HR: 0.392, 95%CI: 0.211–0.726, P = 0.003), endocrine therapy (HR: 0.189, 95%CI: 0.106–0.340, P < 0.0001) were the potential prognostic factors (Table 5).

**Table 4:** Univariate and multivariate COX regression models analyses for the prediction of DFS in breast cancer.

Variables			Univariate				Multivariate		
		P	HR	95 % CI		P	HR	95 % CI	
				Low	High			Low	High
TOX	Low	0.000	1(Ref.)			0.001	1(Ref.)		
	High		0.257	0.140	0.471		0.412	0.248	0.684
Age	<51	0.241	1(Ref.)						
	≥51		1.737	0.690	4.373				
BMI	<23.8	0.301	1(Ref.)						
	≥23.8		0.726	0.395	1.333				
Family history	No	0.061	1(Ref.)						
	Yes		1.934	0.970	3.858				
Basic disease	No	0.427	1(Ref.)						
	Yes		0.752	0.372	1.520				
Menarche age	<15	0.446	1(Ref.)						
	≥15		1.285	0.675	2.446				
Menopause	No	0.869	1(Ref.)						
	Yes		0.924	0.359	2.376				
CA153	<9.82	0.078	1(Ref.)						
	≥9.82		0.578	0.315	1.062				
CEA	<1.49	0.159	1(Ref.)						
	≥1.49		1.651	0.821	3.318				
D-D	<0.25	0.808	1(Ref.)						
	≥0.25		1.075	0.601	1.921				
FBG	<2.6	0.219	1(Ref.)						
	≥2.6		1.449	0.802	2.616				
Neutrophil	<3.23	0.121	1(Ref.)						
	≥3.23		1.746	0.862	3.533				
Lymphocyte	<1.70	0.690	1(Ref.)						
	≥1.70		0.881	0.473	1.640				
Monocyte	<0.35	0.396	1(Ref.)						
	≥0.35		1.350	0.675	2.698				
Type of surgery	Mastectomy	0.378	1(Ref.)						
	Breast-conserving surgery		2.325	0.357	15.141				
Tumor size	≤2	0.683	1(Ref.)						
	>2 and < 5	0.476	1.283	0.646	2.549				
	≥5	0.443	1.639	0.463	5.802				
Pathological TNM Stage	I	0.000	1(Ref.)			0.000	1(Ref.)		
	II	0.638	1.242	0.504	3.061	0.578	1.232	0.590	2.571
	III	0.000	8.603	2.761	26.801	0.000	6.305	3.053	13.020
Molecular subtype	Luminal A	0.089	1(Ref.)						
	Luminal B HER2+	0.042	4.738	1.057	21.244				
	Luminal B HER2-	0.221	2.459	0.583	10.376				
	HER2 enriched	0.445	1.928	0.358	10.390				
	Triple negative	0.675	1.436	0.264	7.794				
E-cad	Negative	0.390	1(Ref.)						
	Positive		2.008	0.409	9.854				
P120	Negative	0.693	1(Ref.)						
	Positive		1.204	0.479	3.026				
P53	Negative	0.550	1(Ref.)						
	Positive		1.156	0.719	1.859				
Blood vessel invasion	No	0.368	1(Ref.)						
	Yes		0.571	0.168	1.934				
Lung metastasis	No	0.013	1(Ref.)			0.010	1(Ref.)		
	Yes		3.268	1.287	8.295		2.855	1.279	6.374
Bone metastasis	No	0.002	1(Ref.)			0.000	1(Ref.)		
	Yes		3.960	1.646	9.526		4.314	2.022	9.204
Liver metastasis	No	0.318	1(Ref.)						
	Yes		1.620	0.629	4.175				
Brain metastasis	No	0.000	1(Ref.)			0.000	1(Ref.)		
	Yes		7.507	2.668	21.126		6.810	3.150	14.723
Chemotherapy	No	0.000	1(Ref.)			0.000	1(Ref.)		
	Yes		0.177	0.087	0.362		0.163	0.079	0.337
Radiotherapy	No	0.009	1(Ref.)			0.002	1(Ref.)		
	Yes		0.275	0.105	0.720		0.324	0.157	0.668
Endocrine therapy	No	0.000	1(Ref.)			0.000	1(Ref.)		
	Yes		0.103	0.033	0.320		0.138	0.073	0.260
Targeted therapy	No	0.652	1(Ref.)						
	Yes		0.781	0.267	2.285

**Table 5:** Univariate and multivariate COX regression models analyses for the prediction of OS in breast cancer.

Variables			Univariate				Multivariate		
		P	HR	95 % CI		P	HR	95 % CI	
				Low	High			Low	High
TOX	Low	0.000	1(Ref.)			0.000	1(Ref.)		
	High		0.294	0.161	0.538		0.395	0.237	0.660
Age	<51	0.651	1(Ref.)						
	≥51		1.227	0.506	2.975				
BMI	<23.8	0.941	1(Ref.)						
	≥23.8		0.978	0.537	1.779				
Family history	No	0.626	1(Ref.)						
	Yes		1.191	0.590	2.401				
Basic disease	No	0.870	1(Ref.)						
	Yes		1.057	0.540	2.070				
Menarche age	<15	0.841	1(Ref.)						
	≥15		0.937	0.498	1.763				
Menopause	No	0.690	1(Ref.)						
	Yes		1.206	0.480	3.027				
CA153	<9.82	0.093	1(Ref.)						
	≥9.82		0.599	0.330	1.089				
CEA	<1.49	0.222	1(Ref.)						
	≥1.49		1.520	0.776	2.976				
D-D	<0.25	0.618	1(Ref.)						
	≥0.25		1.160	0.648	2.076				
FBG	<2.6	0.376	1(Ref.)						
	≥2.6		1.323	0.712	2.459				
Neutrophil	<3.23	0.289	1(Ref.)						
	≥3.23		1.470	0.721	2.995				
Lymphocyte	<1.70	0.575	1(Ref.)						
	≥1.70		0.845	0.469	1.522				
Monocyte	<0.35	0.282	1(Ref.)						
	≥0.35		1.472	0.728	2.975				
Type of surgery	Mastectomy	0.123	1(Ref.)						
	Breast-conserving surgery		0.149	0.013	1.679				
Tumor size	≤2	0.761	1(Ref.)						
	>2 and < 5	0.480	1.282	0.644	2.551				
	≥5	0.619	1.363	0.402	4.623				
Pathological TNM Stage	I	0.000	1(Ref.)			0.000	1(Ref.)		
	II	0.869	1.082	0.423	2.773	0.109	1.860	0.870	3.975
	III	0.001	7.017	2.324	21.188	0.000	8.578	3.973	18.524
Molecular subtype	Luminal A	0.052	1(Ref.)						
	Luminal B HER2+	0.034	4.965	1.132	21.773				
	Luminal B HER2-	0.134	2.890	0.722	11.569				
	HER2 enriched	0.247	2.627	0.512	13.485				
	Triple negative	0.742	1.329	0.244	7.248				
E-cad	Negative	0.216	1(Ref.)						
	Positive		2.866	0.540	15.205				
P120	Negative	0.582	1(Ref.)						
	Positive		1.317	0.495	3.502				
P53	Negative	0.272	1(Ref.)						
	Positive		0.697	0.366	1.326				
Blood vessel invasion	No	0.639	1(Ref.)						
	Yes		0.785	0.285	2.159				
Lung metastasis	No	0.000	1(Ref.)			0.007	1(Ref.)		
	Yes		3.922	2.252	6.830		2.425	1.279	4.597
Bone metastasis	No	0.069	1(Ref.)						
	Yes		2.509	0.939	5.505				
Liver metastasis	No	0.172	1(Ref.)			0.000	1(Ref.)		
	Yes		1.883	0.760	4.664		3.558	1.905	6.646
Brain metastasis	No	0.326	1(Ref.)						
	Yes		1.644	0.610	4.432				
Chemotherapy	No	0.010	1(Ref.)			0.000	1(Ref.)		
	Yes		0.262	0.095	0.726		0.139	0.066	0.290
Radiotherapy	No	0.006	1(Ref.)			0.003	1(Ref.)		
	Yes		0.295	0.123	0.707		0.392	0.211	0.726
Endocrine therapy	No	0.000	1(Ref.)			0.000	1(Ref.)		
	Yes		0.127	0.041	0.393		0.189	0.106	0.340
Targeted therapy	No	0.386	1(Ref.)						
	Yes		0.614	0.204	1.849				

### 3.8 Nomograms established for the prediction of survival outcomes

**Fig. 2:**
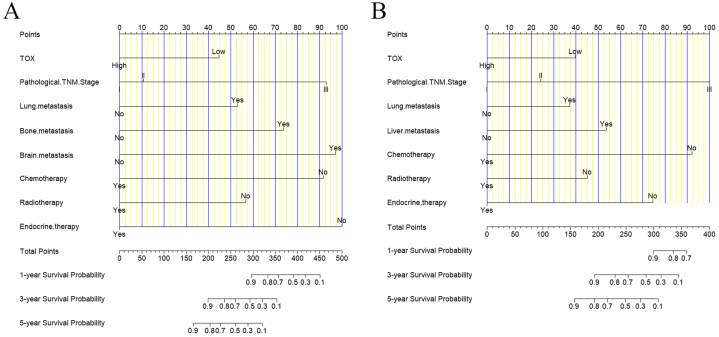
Nomograms established for the prediction of survival outcomes of breast cancer patients. Nomograms for (A) disease free survival and (B) overall survival.

**Fig. 3:**
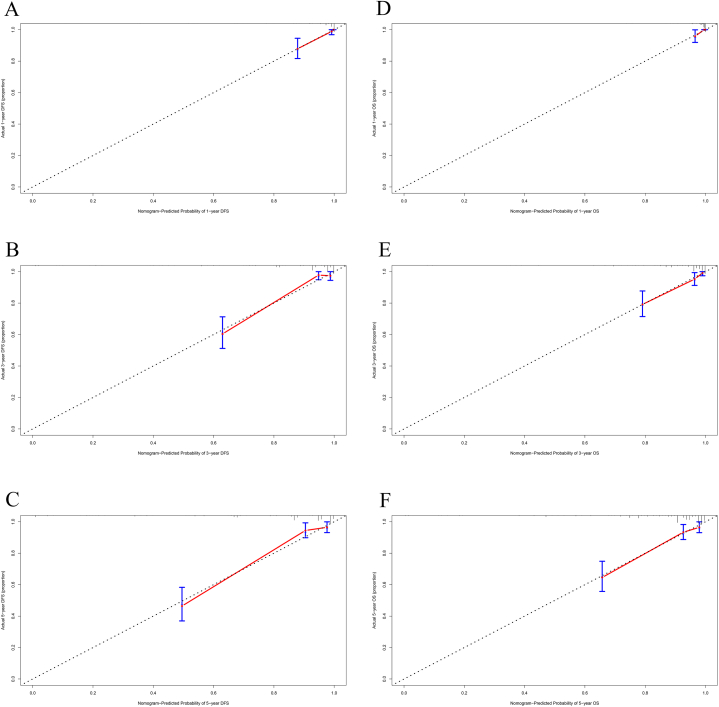
Calibration curves for assessing the performance of nomograms for survival time after surgery for breast cancer patients. Calibration curves for 1-year, 3-year and 5-year survival rate of DFS (A–C), and 1-year, 3-year and 5-year survival rate of OS (D–F).

**Fig. 4:**
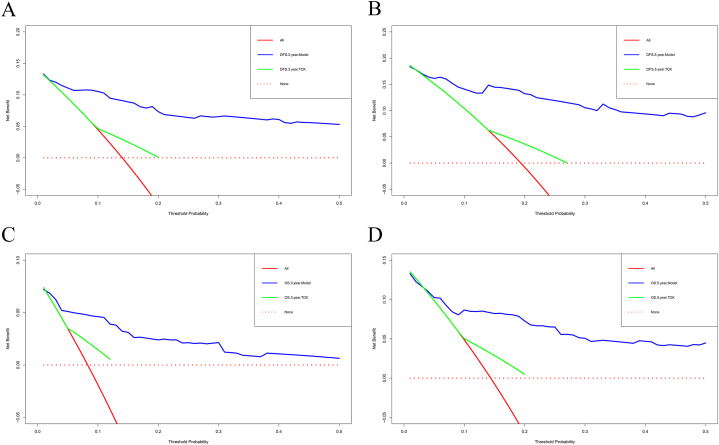
Decision curve analysis for evaluating the clinical utility of the nomograms for survival time after surgery for breast cancer patients. Decision curve analysis for 3-year and 5-year survival rate of DFS (A–B), and 3-year and 5-year survival rate of OS (C–D).

**Fig. 5:**
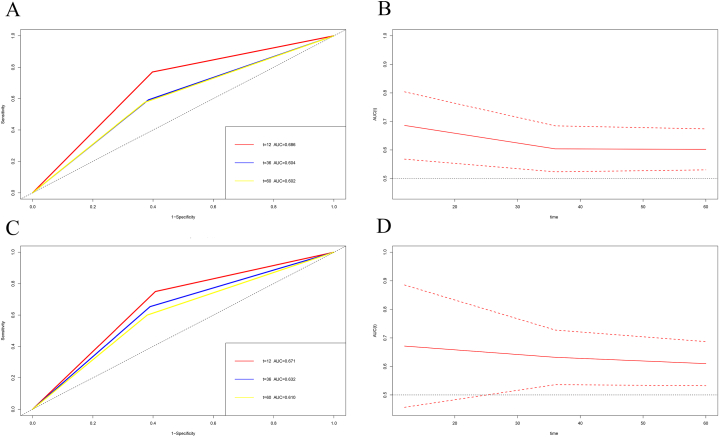
Time-dependent receiver operating characteristic (TDROC) analyzed the plots of area under the receiver operating characteristic curves (AUROCs) for TOX expression in breast cancer patients after surgery of follow-up. A, C) Time-dependent AUROCs for DFS and OS; B, D) 95%CI changes of AUROCs for DFS and OS.

The variables identified by multivariate analyses for DFS, including TOX, pathological TNM Stage, lung metastasis, bone metastasis, brain metastasis, chemotherapy, radiotherapy, endocrine therapy was applied to construct a DFS-predicting nomogram (Fig. 2A). The parameters identified by multivariate analyses for OS, including TOX, pathological TNM Stage, lung metastasis, liver metastasis, chemotherapy, radiotherapy, endocrine therapy was used to establish an OS-predicting nomogram (Fig. 2B). The calibration curves indicated that the prediction line matched well with the reference lines for 1-year, 3-year and 5-year survival rate of DFS (Fig. 3A–C) and OS (Fig. 3D–F). Besides, the decision curve analysis shown that the established nomogram models had so much the better predictive clinical utility compared with TOX alone for the 3-year and 5-year survival rate of DFS (Fig. 4A and B) and OS (Fig. 4C and D). Furthermore, TDROC also performed that AUROC at 1-year survival rate of DFS and OS after surgery of follow-up were 0.686 (95%CI: 0.568–0.804) and 0.671 (95%CI: 0.457–0.885); AUROC at 3-year survival rate of DFS and OS after surgery of follow-up were 0.604 (95%CI: 0.523–0.684) and 0.632 (95%CI: 0.536–0.728); AUROC at 5-year survival rate of DFS and OS after surgery of follow-up were 0.602 (95%CI: 0.530–0.674) and 0.610 (95%CI: 0.532–0.687) (Fig. 5).

## 4 Discussion

TOX has emerged as a prominent regulatory factor for T cell dysfunction in malignant tumors in recent years [25]. More evidence suggests that TOX has different roles in dysfunctional and exhausted T cells, and the levels of TOX expression may be crucial in influencing the function and fate of T cells [26,27]. Some studies have also indicated that the expression of TOX could predict prognosis in patients with malignancy [28]. Li et al. revealed that TOX was expressed both in tumor cells and CD8 T cells, and ovarian cancer patients with high expression of TOX typically had shorter DFS and OS [29]. Besides, the expression of TOX was an independent predictor for ovarian cancer patients by multivariate Cox model analysis [29]. Guo et al. demonstrated that TOX correlated with poor prognosis in lung adenocarcinoma, and influenced the survival of early-stage lung adenocarcinoma, ever-smoking, and low tumor mutation burden (TMB) status [30]. Furthermore, an increase in TOX expression was positively associated with high levels of immune infiltration in functional T cells, such as exhausted T cells [30]. In McGirt LY's study, TOX served as a diagnostic marker in mycosis fungoides and decreased in response to cutaneous T-cell lymphomas therapeutics [31]. In addition, the simultaneous increase of TOX, along with CD244, PD-1, and Tim-3, in T cells was found to correlate with T cell exhaustion in acute myeloid leukemia [32]. TOX might be considered a potential target for reversing T cell exhaustion in acute myeloid leukemia [32]. However, there has been limited research on the relationship between TOX expression and prognosis in breast cancer. Therefore, we elucidated that TOX could serve as a potential predictor for breast cancer prognosis and provide novel insights for understanding the correlation between TOX and breast cancer treatment.

In the current work, we examined the expression of TOX in breast cancer specimens resected from 313 cases by immunohistochemistry. The results indicated that the average expression of TOX in breast cancer tissues was significantly higher than that in normal adjacent tissues. Besides, we also confirmed that the expression of TOX was concerned with operative time, liver metastasis, CA153 levels, and D-D levels. We demonstrated that the low expression of TOX predicted a decrease in DFS and OS. Importantly, multivariate Cox analysis revealed that TOX was a potential independent predictor of DFS and OS. We also analyzed the associations between the expression of TOX and adjuvant therapy after surgery in breast cancer patients. The results indicated that patients with high expression of TOX had longer survival time than those with low TOX expression, especially in patients who received chemotherapy. Wang et al. demonstrated that down-regulating TOX expression in CD8^+^ T cells exerts synergistic effects with anti-PD1 therapy, which is a promising immunotherapy strategy [22]. Chen et al. showed that TOX was related to lactate production and implicated in CD8^+^ T cell dysfunction, and patients with acute myeloid leukemia after chemotherapy had markedly lower lactate concentrations, reducing PD-1 expression, CD8^+^ TEM cells [33]. They demonstrated that targeting TOX gene in acute myeloid leukemia patients could be a meaningful and precise treatment strategy [33]. As is known to all, TNM stage is the remarkable predictor for malignant tumors. We further analyzed the associations between the expression of TOX and TNM stage after surgery in breast cancer patients. The results demonstrated that patients with high expression of TOX had longer survival time than those patients with low expression of TOX, especially in patients with stage II.

The potential mechanisms previously discovered may explain the findings of TOX in breast cancer. Gene silencing via hypermethylation of the abnormal promoter CpG island is a common epigenetic abnormality observed in breast cancer [34]. In a genome-wide comparison of DNA methylation, the promoter CpG island of TOX is highly methylated in 43 % of breast tumors, while it is unmethylated in distant normal breast tissue [35]. The gene mutation or loss of heterozygosity also influences deregulation of TOX gene expression. TOX itself undergoes mutations, which subsequently lead to its abnormal expression. In a mutation screening study of TOX, 4 mutations were found in six primary tumors out of 133 breast cancer tissues [36].

This study has several limitations. Firstly, it was performed as a single-center and retrospective study. Therefore, more patients enrolled with multi-center researches are required to validate our findings. Secondly, heterogeneity among breast cancer patients after surgery may influence the expression of TOX and, consequently, cancer prognosis.

## 5 Conclusions

In conclusion, we verified the expression of TOX in 313 breast cancer patients and elucidated that TOX could predict survival and prognosis. The constructed nomograms incorporating TOX may facilitate decision-making, but require further research. Further studies are required to elucidate the prospective mechanism by which TOX affects the function of tumor cell in breast cancer microenvironment. We hope that the expression level of TOX in breast cancer may provide a novel biomarker for guiding treatment strategies for breast cancer patients.

## CRediT authorship contribution statement

**Chunlei Tan:** Writing – review & editing, Writing – original draft, Resources, Investigation, Funding acquisition, Formal analysis, Data curation, Conceptualization. **Danping Wu:** Formal analysis. **Xiaotian Yang:** Formal analysis. **Shiyuan Zhang:** Investigation. **Shuqiang Liu:** Methodology. **Boqian Yu:** Supervision. **Xiao Yu:** Writing – original draft. **Yuting Xiu:** Writing – review & editing. **Yuanxi Huang:** Resources, Project administration, Data curation, Conceptualization.

## Ethics approval and consent statement

This study was performed in line with the principles of the Declaration of Helsinki. All patients provided written informed consent to participate in the study and for their data to be published. This study was reviewed and approved by the Ethics Committee of Harbin Medical University Cancer Hospital with the approval number: KY2023-38, dated 13, April 2023. We employed a retrospective study, which followed the STROBE guidelines for reporting.

## Data availability statement

The material supporting the conclusion of this article has been included within the article.

## Funding statement

This work was supported by grants from the Practice and Innovation Project for Postgraduates of 10.13039/100010722Harbin Medical University (Grant No. YJSCX2023-79HYD).

## Declaration of competing interest

The authors declare that they have no known competing financial interests or personal relationships that could have appeared to influence the work reported in this paper.

## References

[1] Siegel R.L., Miller K.D., Fuchs H.E., Jemal A. (2021). Cancer Statistics. CA A Cancer J. Clin..

[2] Sung H., Ferlay J., Siegel R.L., Laversanne M., Soerjomataram I., Jemal A., Bray F. (2021). Global cancer Statistics 2020: GLOBOCAN estimates of incidence and mortality worldwide for 36 cancers in 185 countries. CA A Cancer J. Clin..

[3] Sørlie T., Perou C.M., Tibshirani R., Aas T., Geisler S., Johnsen H., Hastie T., Eisen M.B., van de Rijn M., Jeffrey S.S., Thorsen T., Quist H., Matese J.C., Brown P.O., Botstein D., Lønning P.E., Børresen-Dale A.L. (2001). Gene expression patterns of breast carcinomas distinguish tumor subclasses with clinical implications. Proc. Natl. Acad. Sci. U. S. A..

[4] Ciriello G., Gatza M.L., Beck A.H., Wilkerson M.D., Rhie S.K., Pastore A., Zhang H., McLellan M., Yau C., Kandoth C., Bowlby R., Shen H., Hayat S., Fieldhouse R., Lester S.C., Tse G.M.K., Factor R.E., Collins L.C., Allison K.H., Chen Y.-Y., Jensen K., Johnson N.B., Oesterreich S., Mills G.B., Cherniack A.D., Robertson G., Benz C., Sander C., Laird P.W., Hoadley K.A., King T.A., Research Network T.C.G.A., Perou C.M. (2015). Comprehensive molecular portraits of invasive lobular breast cancer. Cell.

[5] Pan H., Gray R., Braybrooke J., Davies C., Taylor C., McGale P., Peto R., Pritchard K.I., Bergh J., Dowsett M., Hayes D.F. (2017). EBCTCG, 20-year risks of breast-cancer recurrence after stopping endocrine therapy at 5 years. N. Engl. J. Med..

[6] Liang Y., Zhang H., Song X., Yang Q. (2020). Metastatic heterogeneity of breast cancer: molecular mechanism and potential therapeutic targets. Semin. Cancer Biol..

[7] Salgado R., Loi S. (2018). Tumour infiltrating lymphocytes in breast cancer: increasing clinical relevance. Lancet Oncol..

[8] Glajcar A., Szpor J., Hodorowicz-Zaniewska D., Tyrak K.E., Okoń K. (2019). The composition of T cell infiltrates varies in primary invasive breast cancer of different molecular subtypes as well as according to tumor size and nodal status. Virchows Arch..

[9] Gajewski T.F., Schreiber H., Fu Y.-X. (2013). Innate and adaptive immune cells in the tumor microenvironment. Nat. Immunol..

[10] Wu Q., Tao X., Luo Y., Zheng S., Lin N., Xie X. (2023). A novel super-enhancer-related gene signature predicts prognosis and immune microenvironment for breast cancer. BMC Cancer.

[11] Zhang Q., Gao C., Shao J., Wang Z. (2021). TIGIT-related transcriptome profile and its association with tumor immune microenvironment in breast cancer. Biosci. Rep..

[12] Bagchi S., Yuan R., Engleman E.G. (2021). Immune checkpoint inhibitors for the treatment of cancer: clinical impact and mechanisms of response and resistance. Annu. Rev. Pathol..

[13] Zhang Y., Chen H., Mo H., Hu X., Gao R., Zhao Y., Liu B., Niu L., Sun X., Yu X., Wang Y., Chang Q., Gong T., Guan X., Hu T., Qian T., Xu B., Ma F., Zhang Z., Liu Z. (2021). Single-cell analyses reveal key immune cell subsets associated with response to PD-L1 blockade in triple-negative breast cancer. Cancer Cell.

[14] Oladejo M., Paulishak W., Wood L. (2023). Synergistic potential of immune checkpoint inhibitors and therapeutic cancer vaccines. Semin. Cancer Biol..

[15] Farshbafnadi M., Pastaki Khoshbin A., Rezaei N. (2021). Immune checkpoint inhibitors for triple-negative breast cancer: from immunological mechanisms to clinical evidence. Int. Immunopharm..

[16] Gaikwad S., Agrawal M.Y., Kaushik I., Ramachandran S., Srivastava S.K. (2022). Immune checkpoint proteins: signaling mechanisms and molecular interactions in cancer immunotherapy. Semin. Cancer Biol..

[17] Budimir N., Thomas G.D., Dolina J.S., Salek-Ardakani S. (2022). Reversing T-cell exhaustion in cancer: lessons learned from PD-1/PD-L1 immune checkpoint blockade. Cancer Immunol. Res..

[18] Han J., Wan M., Ma Z., He P. (2022). The TOX subfamily: all-round players in the immune system. Clin. Exp. Immunol..

[19] Scott A.C., Dündar F., Zumbo P., Chandran S.S., Klebanoff C.A., Shakiba M., Trivedi P., Menocal L., Appleby H., Camara S., Zamarin D., Walther T., Snyder A., Femia M.R., Comen E.A., Wen H.Y., Hellmann M.D., Anandasabapathy N., Liu Y., Altorki N.K., Lauer P., Levy O., Glickman M.S., Kaye J., Betel D., Philip M., Schietinger A. (2019). TOX is a critical regulator of tumour-specific T cell differentiation. Nature.

[20] Yao C., Sun H.-W., Lacey N.E., Ji Y., Moseman E.A., Shih H.-Y., Heuston E.F., Kirby M., Anderson S., Cheng J., Khan O., Handon R., Reilley J., Fioravanti J., Hu J., Gossa S., Wherry E.J., Gattinoni L., McGavern D.B., O'Shea J.J., Schwartzberg P.L., Wu T. (2019). Single-cell RNA-seq reveals TOX as a key regulator of CD8+ T cell persistence in chronic infection. Nat. Immunol..

[21] Khan O., Giles J.R., McDonald S., Manne S., Ngiow S.F., Patel K.P., Werner M.T., Huang A.C., Alexander K.A., Wu J.E., Attanasio J., Yan P., George S.M., Bengsch B., Staupe R.P., Donahue G., Xu W., Amaravadi R.K., Xu X., Karakousis G.C., Mitchell T.C., Schuchter L.M., Kaye J., Berger S.L., Wherry E.J. (2019). TOX transcriptionally and epigenetically programs CD8+ T cell exhaustion. Nature.

[22] Wang X., He Q., Shen H., Xia A., Tian W., Yu W., Sun B. (2019). TOX promotes the exhaustion of antitumor CD8+ T cells by preventing PD1 degradation in hepatocellular carcinoma. J. Hepatol..

[23] Han H.S., Jeong S., Kim H., Kim H.-D., Kim A.R., Kwon M., Park S.-H., Woo C.G., Kim H.K., Lee K.H., Seo S.P., Kang H.W., Kim W.T., Kim W.-J., Yun S.J., Shin E.-C. (2021). TOX-expressing terminally exhausted tumor-infiltrating CD8+ T cells are reinvigorated by co-blockade of PD-1 and TIGIT in bladder cancer. Cancer Lett..

[24] Yang M., Huang Q., Li C., Jiang Z., Sun J., Wang Z., Liang R., Li D., Li B., Zhao H. (2021). TOX acts as a tumor suppressor by inhibiting mTOR signaling in colorectal cancer. Front. Immunol..

[25] Alfei F., Kanev K., Hofmann M., Wu M., Ghoneim H.E., Roelli P., Utzschneider D.T., von Hoesslin M., Cullen J.G., Fan Y., Eisenberg V., Wohlleber D., Steiger K., Merkler D., Delorenzi M., Knolle P.A., Cohen C.J., Thimme R., Youngblood B., Zehn D. (2019). TOX reinforces the phenotype and longevity of exhausted T cells in chronic viral infection. Nature.

[26] Beltra J.-C., Abdel-Hakeem M.S., Manne S., Zhang Z., Huang H., Kurachi M., Su L., Picton L., Ngiow S.F., Muroyama Y., Casella V., Huang Y.J., Giles J.R., Mathew D., Belman J., Klapholz M., Decaluwe H., Huang A.C., Berger S.L., Garcia K.C., Wherry E.J. (2023). Stat 5 opposes the transcription factor Tox and rewires exhausted CD8+ T cells toward durable effector-like states during chronic antigen exposure. Immunity.

[27] Page N., Lemeille S., Vincenti I., Klimek B., Mariotte A., Wagner I., Di Liberto G., Kaye J., Merkler D. (2021). Persistence of self-reactive CD8+ T cells in the CNS requires TOX-dependent chromatin remodeling. Nat. Commun..

[28] Yu X., Li Z. (2015). TOX gene: a novel target for human cancer gene therapy. Am. J. Cancer Res..

[29] Li S., Yang S., Hong Y. (2022). Higher thymocyte selection-associated high mobility group box (TOX) expression predicts poor prognosis in patients with ovarian cancer. BMC Cancer.

[30] Guo L., Li X., Liu R., Chen Y., Ren C., Du S. (2020). TOX correlates with prognosis, immune infiltration, and T cells exhaustion in lung adenocarcinoma. Cancer Med..

[31] McGirt L.Y., Degesys C.A., Johnson V.E., Zic J.A., Zwerner J.P., Eischen C.M. (2016). TOX expression and role in CTCL. J. Eur. Acad. Dermatol. Venereol..

[32] Huang S., Liang C., Zhao Y., Deng T., Tan J., Zha X., Li Y., Chen S. (2022). Increased TOX expression concurrent with PD-1, Tim-3, and CD244 expression in T cells from patients with acute myeloid leukemia. Cytometry B Clin Cytom.

[33] Chen Y., Feng Z., Kuang X., Zhao P., Chen B., Fang Q., Cheng W., Wang J. (2021). Increased lactate in AML blasts upregulates TOX expression, leading to exhaustion of CD8+ cytolytic T cells. Am. J. Cancer Res..

[34] Han Y.-J., Zhang J., Zheng Y., Huo D., Olopade O.I. (2016). Genetic and epigenetic regulation of TOX3 expression in breast cancer. PLoS One.

[35] Tessema M., Yingling C.M., Grimes M.J., Thomas C.L., Liu Y., Leng S., Joste N., Belinsky S.A. (2012). Differential epigenetic regulation of TOX subfamily high mobility group box genes in lung and breast cancers. PLoS One.

[36] Jones J.O., Chin S.-F., Wong-Taylor L.-A., Leaford D., Ponder B.A.J., Caldas C., Maia A.-T. (2013). TOX3 mutations in breast cancer. PLoS One.

