# Supplementary material for: Low expression of TOX predicts poor prognosis of patients with breast cancer in the real world: A retrospective study
Source: Heliyon. 2024 Dec 12;11(1):e41180. doi: 10.1016/j.heliyon.2024.e41180 (PMC11699305; doi:10.1016/j.heliyon.2024.e41180)

**Table S1** Demographic and clinicopathologic characteristics of patients with breast cancer received chemotherapy

|  | level | Overall | Low TOX | High TOX | p |
| --- | --- | --- | --- | --- | --- |
| n |  | 290 | 118 | 172 |  |
| Age | <51 | 148 (51.0) | 65 (55.1) | 83 (48.3) | 0.306 |
|  | ≥51 | 142 (49.0) | 53 (44.9) | 89 (51.7) |  |
| Weight | <62 | 137 (47.2) | 61 (51.7) | 76 (44.2) | 0.255 |
|  | ≥62 | 153 (52.8) | 57 (48.3) | 96 (55.8) |  |
| Height | <1.60 | 98 (33.8) | 43 (36.4) | 55 (32.0) | 0.507 |
|  | ≥1.60 | 192 (66.2) | 75 (63.6) | 117 (68.0) |  |
| BMI | <23.8 | 144 (49.7) | 60 (50.8) | 84 (48.8) | 0.828 |
|  | ≥23.8 | 146 (50.3) | 58 (49.2) | 88 (51.2) |  |
| Family history | No | 230 (79.3) | 91 (77.1) | 139 (80.8) | 0.538 |
|  | Yes | 60 (20.7) | 27 (22.9) | 33 (19.2) |  |
| Basic disease | No | 228 (78.6) | 89 (75.4) | 139 (80.8) | 0.340 |
|  | Yes | 62 (21.4) | 29 (24.6) | 33 (19.2) |  |
| Hypertension | No | 254 (87.6) | 99 (83.9) | 155 (90.1) | 0.163 |
|  | Yes | 36 (12.4) | 19 (16.1) | 17 (9.9) |  |
| Diabetes mellitus | No | 274 (94.5) | 112 (94.9) | 162 (94.2) | 0.996 |
|  | Yes | 16 (5.5) | 6 (5.1) | 10 (5.8) |  |
| Coronary heart disease | No | 279 (96.2) | 113 (95.8) | 166 (96.5) | 0.988 |
|  | Yes | 11 (3.8) | 5 (4.2) | 6 (3.5) |  |
| Menarche age | <15 | 116 (40.0) | 49 (41.5) | 67 (39.0) | 0.751 |
|  | ≥15 | 174 (60.0) | 69 (58.5) | 105 (61.0) |  |
| Menopause | No | 145 (50.0) | 62 (52.5) | 83 (48.3) | 0.550 |
|  | Yes | 145 (50.0) | 56 (47.5) | 89 (51.7) |  |
| ALT | <21 | 130 (44.8) | 49 (41.5) | 81 (47.1) | 0.414 |
|  | ≥21 | 160 (55.2) | 69 (58.5) | 91 (52.9) |  |
| AST | <23 | 136 (46.9) | 50 (42.4) | 86 (50.0) | 0.247 |
|  | ≥23 | 154 (53.1) | 68 (57.6) | 86 (50.0) |  |
| AST/ALT | <1.1 | 143 (49.3) | 61 (51.7) | 82 (47.7) | 0.580 |
|  | ≥1.1 | 147 (50.7) | 57 (48.3) | 90 (52.3) |  |
| LDH | <170 | 146 (50.3) | 60 (50.8) | 86 (50.0) | 0.982 |
|  | ≥170 | 144 (49.7) | 58 (49.2) | 86 (50.0) |  |
| GGT | <14 | 131 (45.2) | 49 (41.5) | 82 (47.7) | 0.361 |
|  | ≥14 | 159 (54.8) | 69 (58.5) | 90 (52.3) |  |
| ALP | <70 | 144 (49.7) | 58 (49.2) | 86 (50.0) | 0.982 |
|  | ≥70 | 146 (50.3) | 60 (50.8) | 86 (50.0) |  |
| GLU | <5.1 | 142 (49.0) | 61 (51.7) | 81 (47.1) | 0.515 |
|  | ≥5.1 | 148 (51.0) | 57 (48.3) | 91 (52.9) |  |
| ALB | <45 | 134 (46.2) | 54 (45.8) | 80 (46.5) | 0.995 |
|  | ≥45 | 156 (53.8) | 64 (54.2) | 92 (53.5) |  |
| BUN | <4.9 | 140 (48.3) | 62 (52.5) | 78 (45.3) | 0.278 |
|  | ≥4.9 | 150 (51.7) | 56 (47.5) | 94 (54.7) |  |
| UR/CR | <0.078 | 140 (48.3) | 61 (51.7) | 79 (45.9) | 0.398 |
|  | ≥0.078 | 150 (51.7) | 57 (48.3) | 93 (54.1) |  |
| CRE | <63 | 143 (49.3) | 58 (49.2) | 85 (49.4) | 1.000 |
|  | ≥63 | 147 (50.7) | 60 (50.8) | 87 (50.6) |  |
| URIC | <253 | 147 (50.7) | 58 (49.2) | 89 (51.7) | 0.753 |
|  | ≥253 | 143 (49.3) | 60 (50.8) | 83 (48.3) |  |
| TBIL | <12.45 | 143 (49.3) | 51 (43.2) | 92 (53.5) | 0.110 |
|  | ≥12.45 | 147 (50.7) | 67 (56.8) | 80 (46.5) |  |
| DBIL | <3.9 | 142 (49.0) | 50 (42.4) | 92 (53.5) | 0.082 |
|  | ≥3.9 | 148 (51.0) | 68 (57.6) | 80 (46.5) |  |
| IBIL | <8.29 | 143 (49.3) | 50 (42.4) | 93 (54.1) | 0.066 |
|  | ≥8.29 | 147 (50.7) | 68 (57.6) | 79 (45.9) |  |
| TP | <74 | 124 (42.8) | 51 (43.2) | 73 (42.4) | 0.991 |
|  | ≥74 | 166 (57.2) | 67 (56.8) | 99 (57.6) |  |
| G | <29 | 132 (45.5) | 55 (46.6) | 77 (44.8) | 0.850 |
|  | ≥29 | 158 (54.5) | 63 (53.4) | 95 (55.2) |  |
| A/G | <1.5 | 95 (32.8) | 33 (28.0) | 62 (36.0) | 0.189 |
|  | ≥1.5 | 195 (67.2) | 85 (72.0) | 110 (64.0) |  |
| PAB | <267 | 144 (49.7) | 54 (45.8) | 90 (52.3) | 0.328 |
|  | ≥267 | 146 (50.3) | 64 (54.2) | 82 (47.7) |  |
| CO2 | <28.5 | 143 (49.3) | 55 (46.6) | 88 (51.2) | 0.521 |
|  | ≥28.5 | 147 (50.7) | 63 (53.4) | 84 (48.8) |  |
| CA153 | <9.82 | 140 (48.3) | 49 (41.5) | 91 (52.9) | 0.074 |
|  | ≥9.82 | 150 (51.7) | 69 (58.5) | 81 (47.1) |  |
| CEA | <1.49 | 147 (50.7) | 60 (50.8) | 87 (50.6) | 1.000 |
|  | ≥1.49 | 143 (49.3) | 58 (49.2) | 85 (49.4) |  |
| D-D | <0.25 | 144 (49.7) | 67 (56.8) | 77 (44.8) | 0.059 |
|  | ≥0.25 | 146 (50.3) | 51 (43.2) | 95 (55.2) |  |
| FBG | <2.6 | 143 (49.3) | 63 (53.4) | 80 (46.5) | 0.302 |
|  | ≥2.6 | 147 (50.7) | 55 (46.6) | 92 (53.5) |  |
| INR | <0.97 | 132 (45.5) | 53 (44.9) | 79 (45.9) | 0.960 |
|  | ≥0.97 | 158 (54.5) | 65 (55.1) | 93 (54.1) |  |
| PT | <11.1 | 131 (45.2) | 53 (44.9) | 78 (45.3) | 1.000 |
|  | ≥11.1 | 159 (54.8) | 65 (55.1) | 94 (54.7) |  |
| APTT | <27.5 | 139 (47.9) | 58 (49.2) | 81 (47.1) | 0.822 |
|  | ≥27.5 | 151 (52.1) | 60 (50.8) | 91 (52.9) |  |
| TT | <17.2 | 142 (49.0) | 54 (45.8) | 88 (51.2) | 0.433 |
|  | ≥17.2 | 148 (51.0) | 64 (54.2) | 84 (48.8) |  |
| Blood type | A | 65 (22.4) | 22 (18.6) | 43 (25.0) | 0.441 |
|  | B | 112 (38.6) | 44 (37.3) | 68 (39.5) |  |
|  | O | 82 (28.3) | 38 (32.2) | 44 (25.6) |  |
|  | AB | 31 (10.7) | 14 (11.9) | 17 (9.9) |  |
| White blood cell | <5.45 | 145 (50.0) | 58 (49.2) | 87 (50.6) | 0.905 |
|  | ≥5.45 | 145 (50.0) | 60 (50.8) | 85 (49.4) |  |
| Red blood cell | <4.2 | 143 (49.3) | 51 (43.2) | 92 (53.5) | 0.110 |
|  | ≥4.2 | 147 (50.7) | 67 (56.8) | 80 (46.5) |  |
| Hemoglobin | <127 | 141 (48.6) | 51 (43.2) | 90 (52.3) | 0.160 |
|  | ≥127 | 149 (51.4) | 67 (56.8) | 82 (47.7) |  |
| Neutrophil | <3.23 | 143 (49.3) | 57 (48.3) | 86 (50.0) | 0.870 |
|  | ≥3.23 | 147 (50.7) | 61 (51.7) | 86 (50.0) |  |
| Lymphocyte | <1.70 | 145 (50.0) | 56 (47.5) | 89 (51.7) | 0.550 |
|  | ≥1.70 | 145 (50.0) | 62 (52.5) | 83 (48.3) |  |
| Monocyte | <0.35 | 141 (48.6) | 58 (49.2) | 83 (48.3) | 0.976 |
|  | ≥0.35 | 149 (51.4) | 60 (50.8) | 89 (51.7) |  |
| Eosinophil | <0.07 | 140 (48.3) | 56 (47.5) | 84 (48.8) | 0.911 |
|  | ≥0.07 | 150 (51.7) | 62 (52.5) | 88 (51.2) |  |
| Basophil | <0.02 | 135 (46.6) | 50 (42.4) | 85 (49.4) | 0.288 |
|  | ≥0.02 | 155 (53.4) | 68 (57.6) | 87 (50.6) |  |
| Platelet | <233 | 141 (48.6) | 52 (44.1) | 89 (51.7) | 0.244 |
|  | ≥233 | 149 (51.4) | 66 (55.9) | 83 (48.3) |  |
| Primary tumor site | Upper outer quadrant | 164 (56.6) | 68 (57.6) | 96 (55.8) | 0.737 |
|  | Lower outer quadrant | 28 (9.7) | 9 (7.6) | 19 (11.0) |  |
|  | Lower inner quadrant | 20 (6.9) | 10 (8.5) | 10 (5.8) |  |
|  | Upper inner quadrant | 41 (14.1) | 15 (12.7) | 26 (15.1) |  |
|  | Central | 37 (12.8) | 16 (13.6) | 21 (12.2) |  |
| US-BIRADS | BIRADS 4 | 159 (54.8) | 65 (55.1) | 94 (54.7) | 0.654 |
|  | BIRADS 5 | 123 (42.4) | 51 (43.2) | 72 (41.9) |  |
|  | BIRADS 6 | 8 (2.8) | 2 (1.7) | 6 (3.5) |  |
| Loss of appetite | No | 71 (24.5) | 32 (27.1) | 39 (22.7) | 0.468 |
|  | Yes | 219 (75.5) | 86 (72.9) | 133 (77.3) |  |
| Nausea | No | 11 (3.8) | 6 (5.1) | 5 (2.9) | 0.522 |
|  | Yes | 279 (96.2) | 112 (94.9) | 167 (97.1) |  |
| Vomit | No | 190 (65.5) | 79 (66.9) | 111 (64.5) | 0.765 |
|  | Yes | 100 (34.5) | 39 (33.1) | 61 (35.5) |  |
| Diarrhea | No | 276 (95.2) | 110 (93.2) | 166 (96.5) | 0.315 |
|  | Yes | 14 (4.8) | 8 (6.8) | 6 (3.5) |  |
| Oral ulcer | No | 248 (85.5) | 103 (87.3) | 145 (84.3) | 0.589 |
|  | Yes | 42 (14.5) | 15 (12.7) | 27 (15.7) |  |
| Alopecia | No | 21 (7.2) | 9 (7.6) | 12 (7.0) | 1.000 |
|  | Yes | 269 (92.8) | 109 (92.4) | 160 (93.0) |  |
| Peripheral neuropathy | No | 179 (61.7) | 70 (59.3) | 109 (63.4) | 0.566 |
|  | Yes | 111 (38.3) | 48 (40.7) | 63 (36.6) |  |
| Anemia | Grade 0 | 265 (91.4) | 109 (92.4) | 156 (90.7) | 0.365 |
|  | Grade 1-2 | 24 (8.3) | 8 (6.8) | 16 (9.3) |  |
|  | Grade 3-4 | 1 (0.3) | 1 (0.8) | 0 (0.0) |  |
| Leukopenia | Grade 0 | 241 (83.1) | 101 (85.6) | 140 (81.4) | 0.644 |
|  | Grade 1-2 | 46 (15.9) | 16 (13.6) | 30 (17.4) |  |
|  | Grade 3-4 | 3 (1.0) | 1 (0.8) | 2 (1.2) |  |
| Neutropenia | Grade 0 | 254 (87.6) | 104 (88.1) | 150 (87.2) | 0.971 |
|  | Grade 1-2 | 31 (10.7) | 12 (10.2) | 19 (11.0) |  |
|  | Grade 3-4 | 5 (1.7) | 2 (1.7) | 3 (1.7) |  |
| Thrombocytopenia | Grade 0 | 288 (99.3) | 117 (99.2) | 171 (99.4) | 1.000 |
|  | Grade 3-4 | 2 (0.7) | 1 (0.8) | 1 (0.6) |  |
| Gastrointestinal reaction | No | 73 (25.2) | 32 (27.1) | 41 (23.8) | 0.621 |
|  | Yes | 217 (74.8) | 86 (72.9) | 131 (76.2) |  |
| Myelosuppression | No | 186 (64.1) | 80 (67.8) | 106 (61.6) | 0.341 |
|  | Yes | 104 (35.9) | 38 (32.2) | 66 (38.4) |  |
| Hepatic dysfunction | No | 154 (53.1) | 60 (50.8) | 94 (54.7) | 0.605 |
|  | Yes | 136 (46.9) | 58 (49.2) | 78 (45.3) |  |
| Operative time | <75 | 134 (46.2) | 47 (39.8) | 87 (50.6) | 0.092 |
|  | ≥75 | 156 (53.8) | 71 (60.2) | 85 (49.4) |  |
| Type of surgery | Mastectomy | 273 (94.1) | 109 (92.4) | 164 (95.3) | 0.421 |
|  | Breast-conserving surgery | 17 (5.9) | 9 (7.6) | 8 (4.7) |  |
| Tumor size | ≤2 | 137 (47.2) | 61 (51.7) | 76 (44.2) | 0.440 |
|  | ＞2 and＜5 | 144 (49.7) | 54 (45.8) | 90 (52.3) |  |
|  | ≥5 | 9 (3.1) | 3 (2.5) | 6 (3.5) |  |
| Histologic grade | I | 5 (1.7) | 4 (3.4) | 1 (0.6) | 0.246 |
|  | II | 169 (58.3) | 70 (59.3) | 99 (57.6) |  |
|  | III | 101 (34.8) | 37 (31.4) | 64 (37.2) |  |
|  | Unknown | 15 (5.2) | 7 (5.9) | 8 (4.7) |  |
| Pathological T Stage | T1 | 151 (52.1) | 64 (54.2) | 87 (50.6) | 0.898 |
|  | T2 | 128 (44.1) | 50 (42.4) | 78 (45.3) |  |
|  | T3 | 9 (3.1) | 3 (2.5) | 6 (3.5) |  |
|  | T4 | 2 (0.7) | 1 (0.8) | 1 (0.6) |  |
| Pathological N Stage | N0 | 112 (38.6) | 40 (33.9) | 72 (41.9) | 0.121 |
|  | N1 | 94 (32.4) | 38 (32.2) | 56 (32.6) |  |
|  | N2 | 48 (16.6) | 19 (16.1) | 29 (16.9) |  |
|  | N3 | 36 (12.4) | 21 (17.8) | 15 (8.7) |  |
| Pathological TNM Stage | I | 70 (24.1) | 28 (23.7) | 42 (24.4) | 0.313 |
|  | II | 133 (45.9) | 49 (41.5) | 84 (48.8) |  |
|  | III | 87 (30.0) | 41 (34.7) | 46 (26.7) |  |
| TLN | <16 | 134 (46.2) | 60 (50.8) | 74 (43.0) | 0.233 |
|  | ≥16 | 156 (53.8) | 58 (49.2) | 98 (57.0) |  |
| PLN | <1 | 117 (40.3) | 42 (35.6) | 75 (43.6) | 0.213 |
|  | ≥1 | 173 (59.7) | 76 (64.4) | 97 (56.4) |  |
| TALN | <14 | 135 (46.6) | 62 (52.5) | 73 (42.4) | 0.115 |
|  | ≥14 | 155 (53.4) | 56 (47.5) | 99 (57.6) |  |
| PALN | <1 | 139 (47.9) | 52 (44.1) | 87 (50.6) | 0.331 |
|  | ≥1 | 151 (52.1) | 66 (55.9) | 85 (49.4) |  |
| Molecular subtype | Luminal A | 47 (16.2) | 22 (18.6) | 25 (14.5) | 0.894 |
|  | Luminal B HER2+ | 61 (21.0) | 23 (19.5) | 38 (22.1) |  |
|  | Luminal B HER2- | 58 (20.0) | 24 (20.3) | 34 (19.8) |  |
|  | HER2 enriched | 60 (20.7) | 23 (19.5) | 37 (21.5) |  |
|  | Triple negative | 64 (22.1) | 26 (22.0) | 38 (22.1) |  |
| ER | 0-25% | 139 (47.9) | 59 (50.0) | 80 (46.5) | 0.903 |
|  | 26-50% | 24 (8.3) | 10 (8.5) | 14 (8.1) |  |
|  | 51-75% | 44 (15.2) | 18 (15.3) | 26 (15.1) |  |
|  | 76-100% | 83 (28.6) | 31 (26.3) | 52 (30.2) |  |
| PR | 0-25% | 183 (63.1) | 75 (63.6) | 108 (62.8) | 0.295 |
|  | 26-50% | 30 (10.3) | 8 (6.8) | 22 (12.8) |  |
|  | 51-75% | 32 (11.0) | 13 (11.0) | 19 (11.0) |  |
|  | 76-100% | 45 (15.5) | 22 (18.6) | 23 (13.4) |  |
| HER2 | Negative | 169 (58.3) | 72 (61.0) | 97 (56.4) | 0.507 |
|  | Positive | 121 (41.7) | 46 (39.0) | 75 (43.6) |  |
| Ki67 | 0-25% | 130 (44.8) | 62 (52.5) | 68 (39.5) | 0.144 |
|  | 26-50% | 99 (34.1) | 36 (30.5) | 63 (36.6) |  |
|  | 51-75% | 46 (15.9) | 14 (11.9) | 32 (18.6) |  |
|  | 76-100% | 15 (5.2) | 6 (5.1) | 9 (5.2) |  |
| CK5 6 | Negative | 204 (70.3) | 85 (72.0) | 119 (69.2) | 0.696 |
|  | Positive | 86 (29.7) | 33 (28.0) | 53 (30.8) |  |
| Ecad | Negative | 11 (3.8) | 8 (6.8) | 3 (1.7) | 0.058 |
|  | Positive | 279 (96.2) | 110 (93.2) | 169 (98.3) |  |
| P120 | Negative | 275 (94.8) | 109 (92.4) | 166 (96.5) | 0.196 |
|  | Positive | 15 (5.2) | 9 (7.6) | 6 (3.5) |  |
| P53 | Negative | 158 (54.5) | 71 (60.2) | 87 (50.6) | 0.136 |
|  | Positive | 132 (45.5) | 47 (39.8) | 85 (49.4) |  |
| Blood vessel invasion | No | 268 (92.4) | 110 (93.2) | 158 (91.9) | 0.838 |
|  | Yes | 22 (7.6) | 8 (6.8) | 14 (8.1) |  |
| Lung metastasis | No | 259 (89.3) | 104 (88.1) | 155 (90.1) | 0.732 |
|  | Yes | 31 (10.7) | 14 (11.9) | 17 (9.9) |  |
| Bone metastasis | No | 253 (87.2) | 101 (85.6) | 152 (88.4) | 0.605 |
|  | Yes | 37 (12.8) | 17 (14.4) | 20 (11.6) |  |
| Liver metastasis | No | 264 (91.0) | 101 (85.6) | 163 (94.8) | 0.013 |
|  | Yes | 26 (9.0) | 17 (14.4) | 9 (5.2) |  |
| Mediastinal metastasis | No | 277 (95.5) | 110 (93.2) | 167 (97.1) | 0.202 |
|  | Yes | 13 (4.5) | 8 (6.8) | 5 (2.9) |  |
| Brain metastasis | No | 275 (94.8) | 109 (92.4) | 166 (96.5) | 0.196 |
|  | Yes | 15 (5.2) | 9 (7.6) | 6 (3.5) |  |
| Pleural metastasis | No | 279 (96.2) | 113 (95.8) | 166 (96.5) | 0.988 |
|  | Yes | 11 (3.8) | 5 (4.2) | 6 (3.5) |  |
| Chest wall metastasis | No | 280 (96.6) | 114 (96.6) | 166 (96.5) | 1.000 |
|  | Yes | 10 (3.4) | 4 (3.4) | 6 (3.5) |  |
| Axillary metastasis | No | 136 (46.9) | 53 (44.9) | 83 (48.3) | 0.660 |
|  | Yes | 154 (53.1) | 65 (55.1) | 89 (51.7) |  |
| Clavicle metastasis | No | 245 (84.5) | 101 (85.6) | 144 (83.7) | 0.789 |
|  | Yes | 45 (15.5) | 17 (14.4) | 28 (16.3) |  |
| Chemotherapy times | <7 | 150 (51.7) | 63 (53.4) | 87 (50.6) | 0.726 |
|  | ≥7 | 140 (48.3) | 55 (46.6) | 85 (49.4) |  |
| Radiotherapy | No | 200 (69.0) | 77 (65.3) | 123 (71.5) | 0.316 |
|  | Yes | 90 (31.0) | 41 (34.7) | 49 (28.5) |  |
| Endocrine therapy | No | 141 (48.6) | 55 (46.6) | 86 (50.0) | 0.654 |
|  | Yes | 149 (51.4) | 63 (53.4) | 86 (50.0) |  |
| Targeted therapy | No | 256 (88.3) | 102 (86.4) | 154 (89.5) | 0.536 |
|  | Yes | 34 (11.7) | 16 (13.6) | 18 (10.5) |  |

**Table S2** Demographic and clinicopathologic characteristics of patients with breast cancer received endocrine therapy

|  | level | Overall | Low TOX | High TOX | p |
| --- | --- | --- | --- | --- | --- |
| n |  | 163 | 70 | 93 |  |
| Age | <51 | 95 (58.3) | 42 (60.0) | 53 (57.0) | 0.822 |
|  | ≥51 | 68 (41.7) | 28 (40.0) | 40 (43.0) |  |
| Weight | <62 | 78 (47.9) | 32 (45.7) | 46 (49.5) | 0.752 |
|  | ≥62 | 85 (52.1) | 38 (54.3) | 47 (50.5) |  |
| Height | <1.60 | 52 (31.9) | 27 (38.6) | 25 (26.9) | 0.157 |
|  | ≥1.60 | 111 (68.1) | 43 (61.4) | 68 (73.1) |  |
| BMI | <23.8 | 82 (50.3) | 33 (47.1) | 49 (52.7) | 0.587 |
|  | ≥23.8 | 81 (49.7) | 37 (52.9) | 44 (47.3) |  |
| Family history | No | 132 (81.0) | 52 (74.3) | 80 (86.0) | 0.091 |
|  | Yes | 31 (19.0) | 18 (25.7) | 13 (14.0) |  |
| Basic disease | No | 132 (81.0) | 55 (78.6) | 77 (82.8) | 0.632 |
|  | Yes | 31 (19.0) | 15 (21.4) | 16 (17.2) |  |
| Hypertension | No | 145 (89.0) | 61 (87.1) | 84 (90.3) | 0.697 |
|  | Yes | 18 (11.0) | 9 (12.9) | 9 (9.7) |  |
| Diabetes mellitus | No | 159 (97.5) | 69 (98.6) | 90 (96.8) | 0.824 |
|  | Yes | 4 (2.5) | 1 (1.4) | 3 (3.2) |  |
| Coronary heart disease | No | 157 (96.3) | 66 (94.3) | 91 (97.8) | 0.438 |
|  | Yes | 6 (3.7) | 4 (5.7) | 2 (2.2) |  |
| Menarche age | <15 | 64 (39.3) | 29 (41.4) | 35 (37.6) | 0.742 |
|  | ≥15 | 99 (60.7) | 41 (58.6) | 58 (62.4) |  |
| Menopause | No | 95 (58.3) | 43 (61.4) | 52 (55.9) | 0.585 |
|  | Yes | 68 (41.7) | 27 (38.6) | 41 (44.1) |  |
| ALT | <21 | 82 (50.3) | 34 (48.6) | 48 (51.6) | 0.821 |
|  | ≥21 | 81 (49.7) | 36 (51.4) | 45 (48.4) |  |
| AST | <23 | 87 (53.4) | 39 (55.7) | 48 (51.6) | 0.718 |
|  | ≥23 | 76 (46.6) | 31 (44.3) | 45 (48.4) |  |
| AST/ALT | <1.1 | 79 (48.5) | 35 (50.0) | 44 (47.3) | 0.856 |
|  | ≥1.1 | 84 (51.5) | 35 (50.0) | 49 (52.7) |  |
| LDH | <170 | 88 (54.0) | 37 (52.9) | 51 (54.8) | 0.926 |
|  | ≥170 | 75 (46.0) | 33 (47.1) | 42 (45.2) |  |
| GGT | <14 | 85 (52.1) | 35 (50.0) | 50 (53.8) | 0.751 |
|  | ≥14 | 78 (47.9) | 35 (50.0) | 43 (46.2) |  |
| ALP | <70 | 98 (60.1) | 45 (64.3) | 53 (57.0) | 0.435 |
|  | ≥70 | 65 (39.9) | 25 (35.7) | 40 (43.0) |  |
| GLU | <5.1 | 81 (49.7) | 37 (52.9) | 44 (47.3) | 0.587 |
|  | ≥5.1 | 82 (50.3) | 33 (47.1) | 49 (52.7) |  |
| ALB | <45 | 79 (48.5) | 33 (47.1) | 46 (49.5) | 0.893 |
|  | ≥45 | 84 (51.5) | 37 (52.9) | 47 (50.5) |  |
| BUN | <4.9 | 86 (52.8) | 40 (57.1) | 46 (49.5) | 0.416 |
|  | ≥4.9 | 77 (47.2) | 30 (42.9) | 47 (50.5) |  |
| UR/CR | <0.078 | 94 (57.7) | 42 (60.0) | 52 (55.9) | 0.717 |
|  | ≥0.078 | 69 (42.3) | 28 (40.0) | 41 (44.1) |  |
| CRE | <63 | 75 (46.0) | 35 (50.0) | 40 (43.0) | 0.467 |
|  | ≥63 | 88 (54.0) | 35 (50.0) | 53 (57.0) |  |
| URIC | <253 | 77 (47.2) | 32 (45.7) | 45 (48.4) | 0.857 |
|  | ≥253 | 86 (52.8) | 38 (54.3) | 48 (51.6) |  |
| TBIL | <12.45 | 83 (50.9) | 31 (44.3) | 52 (55.9) | 0.190 |
|  | ≥12.45 | 80 (49.1) | 39 (55.7) | 41 (44.1) |  |
| DBIL | <3.9 | 79 (48.5) | 28 (40.0) | 51 (54.8) | 0.086 |
|  | ≥3.9 | 84 (51.5) | 42 (60.0) | 42 (45.2) |  |
| IBIL | <8.29 | 84 (51.5) | 30 (42.9) | 54 (58.1) | 0.078 |
|  | ≥8.29 | 79 (48.5) | 40 (57.1) | 39 (41.9) |  |
| TP | <74 | 70 (42.9) | 28 (40.0) | 42 (45.2) | 0.618 |
|  | ≥74 | 93 (57.1) | 42 (60.0) | 51 (54.8) |  |
| G | <29 | 71 (43.6) | 28 (40.0) | 43 (46.2) | 0.525 |
|  | ≥29 | 92 (56.4) | 42 (60.0) | 50 (53.8) |  |
| A/G | <1.5 | 51 (31.3) | 20 (28.6) | 31 (33.3) | 0.632 |
|  | ≥1.5 | 112 (68.7) | 50 (71.4) | 62 (66.7) |  |
| PAB | <267 | 85 (52.1) | 35 (50.0) | 50 (53.8) | 0.751 |
|  | ≥267 | 78 (47.9) | 35 (50.0) | 43 (46.2) |  |
| CO2 | <28.5 | 85 (52.1) | 36 (51.4) | 49 (52.7) | 0.999 |
|  | ≥28.5 | 78 (47.9) | 34 (48.6) | 44 (47.3) |  |
| CA153 | <9.82 | 83 (50.9) | 33 (47.1) | 50 (53.8) | 0.497 |
|  | ≥9.82 | 80 (49.1) | 37 (52.9) | 43 (46.2) |  |
| CEA | <1.49 | 79 (48.5) | 37 (52.9) | 42 (45.2) | 0.415 |
|  | ≥1.49 | 84 (51.5) | 33 (47.1) | 51 (54.8) |  |
| D-D | <0.25 | 87 (53.4) | 43 (61.4) | 44 (47.3) | 0.103 |
|  | ≥0.25 | 76 (46.6) | 27 (38.6) | 49 (52.7) |  |
| FBG | <2.6 | 90 (55.2) | 42 (60.0) | 48 (51.6) | 0.365 |
|  | ≥2.6 | 73 (44.8) | 28 (40.0) | 45 (48.4) |  |
| INR | <0.97 | 68 (41.7) | 30 (42.9) | 38 (40.9) | 0.924 |
|  | ≥0.97 | 95 (58.3) | 40 (57.1) | 55 (59.1) |  |
| PT | <11.1 | 68 (41.7) | 30 (42.9) | 38 (40.9) | 0.924 |
|  | ≥11.1 | 95 (58.3) | 40 (57.1) | 55 (59.1) |  |
| APTT | <27.5 | 82 (50.3) | 35 (50.0) | 47 (50.5) | 1.000 |
|  | ≥27.5 | 81 (49.7) | 35 (50.0) | 46 (49.5) |  |
| TT | <17.2 | 80 (49.1) | 31 (44.3) | 49 (52.7) | 0.366 |
|  | ≥17.2 | 83 (50.9) | 39 (55.7) | 44 (47.3) |  |
| Blood type | A | 42 (25.8) | 18 (25.7) | 24 (25.8) | 0.981 |
|  | B | 58 (35.6) | 24 (34.3) | 34 (36.6) |  |
|  | O | 44 (27.0) | 20 (28.6) | 24 (25.8) |  |
|  | AB | 19 (11.7) | 8 (11.4) | 11 (11.8) |  |
| White blood cell | <5.45 | 76 (46.6) | 31 (44.3) | 45 (48.4) | 0.718 |
|  | ≥5.45 | 87 (53.4) | 39 (55.7) | 48 (51.6) |  |
| Red blood cell | <4.2 | 92 (56.4) | 33 (47.1) | 59 (63.4) | 0.055 |
|  | ≥4.2 | 71 (43.6) | 37 (52.9) | 34 (36.6) |  |
| Hemoglobin | <127 | 88 (54.0) | 32 (45.7) | 56 (60.2) | 0.093 |
|  | ≥127 | 75 (46.0) | 38 (54.3) | 37 (39.8) |  |
| Neutrophil | <3.23 | 79 (48.5) | 34 (48.6) | 45 (48.4) | 1.000 |
|  | ≥3.23 | 84 (51.5) | 36 (51.4) | 48 (51.6) |  |
| Lymphocyte | <1.70 | 81 (49.7) | 29 (41.4) | 52 (55.9) | 0.094 |
|  | ≥1.70 | 82 (50.3) | 41 (58.6) | 41 (44.1) |  |
| Monocyte | <0.35 | 79 (48.5) | 34 (48.6) | 45 (48.4) | 1.000 |
|  | ≥0.35 | 84 (51.5) | 36 (51.4) | 48 (51.6) |  |
| Eosinophil | <0.07 | 89 (54.6) | 35 (50.0) | 54 (58.1) | 0.387 |
|  | ≥0.07 | 74 (45.4) | 35 (50.0) | 39 (41.9) |  |
| Basophil | <0.02 | 76 (46.6) | 30 (42.9) | 46 (49.5) | 0.498 |
|  | ≥0.02 | 87 (53.4) | 40 (57.1) | 47 (50.5) |  |
| Platelet | <233 | 82 (50.3) | 31 (44.3) | 51 (54.8) | 0.240 |
|  | ≥233 | 81 (49.7) | 39 (55.7) | 42 (45.2) |  |
| Primary tumor site | Upper outer quadrant | 90 (55.2) | 36 (51.4) | 54 (58.1) | 0.020 |
|  | Lower outer quadrant | 18 (11.0) | 4 (5.7) | 14 (15.1) |  |
|  | Lower inner quadrant | 12 (7.4) | 10 (14.3) | 2 (2.2) |  |
|  | Upper inner quadrant | 22 (13.5) | 10 (14.3) | 12 (12.9) |  |
|  | Central | 21 (12.9) | 10 (14.3) | 11 (11.8) |  |
| Operative time | <75 | 73 (44.8) | 24 (34.3) | 49 (52.7) | 0.029 |
|  | ≥75 | 90 (55.2) | 46 (65.7) | 44 (47.3) |  |
| Type of surgery | Mastectomy | 150 (92.0) | 63 (90.0) | 87 (93.5) | 0.592 |
|  | Breast-conserving surgery | 13 (8.0) | 7 (10.0) | 6 (6.5) |  |
| Tumor size | ≤2 | 88 (54.0) | 41 (58.6) | 47 (50.5) | 0.507 |
|  | ＞2 and＜5 | 71 (43.6) | 28 (40.0) | 43 (46.2) |  |
|  | ≥5 | 4 (2.5) | 1 (1.4) | 3 (3.2) |  |
| Histologic grade | I | 8 (4.9) | 4 (5.7) | 4 (4.3) | 0.672 |
|  | II | 122 (74.8) | 49 (70.0) | 73 (78.5) |  |
|  | III | 25 (15.3) | 13 (18.6) | 12 (12.9) |  |
|  | Unknown | 8 (4.9) | 4 (5.7) | 4 (4.3) |  |
| Pathological T Stage | T1 | 97 (59.5) | 45 (64.3) | 52 (55.9) | 0.558 |
|  | T2 | 61 (37.4) | 24 (34.3) | 37 (39.8) |  |
|  | T3 | 4 (2.5) | 1 (1.4) | 3 (3.2) |  |
|  | T4 | 1 (0.6) | 0 (0.0) | 1 (1.1) |  |
| Pathological N Stage | N0 | 57 (35.0) | 19 (27.1) | 38 (40.9) | 0.307 |
|  | N1 | 58 (35.6) | 28 (40.0) | 30 (32.3) |  |
|  | N2 | 27 (16.6) | 12 (17.1) | 15 (16.1) |  |
|  | N3 | 21 (12.9) | 11 (15.7) | 10 (10.8) |  |
| Pathological TNM Stage | I | 43 (26.4) | 17 (24.3) | 26 (28.0) | 0.692 |
|  | II | 72 (44.2) | 30 (42.9) | 42 (45.2) |  |
|  | III | 48 (29.4) | 23 (32.9) | 25 (26.9) |  |
| TLN | <16 | 76 (46.6) | 37 (52.9) | 39 (41.9) | 0.221 |
|  | ≥16 | 87 (53.4) | 33 (47.1) | 54 (58.1) |  |
| PLN | <1 | 59 (36.2) | 20 (28.6) | 39 (41.9) | 0.111 |
|  | ≥1 | 104 (63.8) | 50 (71.4) | 54 (58.1) |  |
| TALN | <14 | 78 (47.9) | 38 (54.3) | 40 (43.0) | 0.205 |
|  | ≥14 | 85 (52.1) | 32 (45.7) | 53 (57.0) |  |
| PALN | <1 | 78 (47.9) | 29 (41.4) | 49 (52.7) | 0.205 |
|  | ≥1 | 85 (52.1) | 41 (58.6) | 44 (47.3) |  |
| Molecular subtype | Luminal A | 56 (34.4) | 27 (38.6) | 29 (31.2) | 0.616 |
|  | Luminal B HER2+ | 50 (30.7) | 20 (28.6) | 30 (32.3) |  |
|  | Luminal B HER2- | 57 (35.0) | 23 (32.9) | 34 (36.6) |  |
| ER | 0-25% | 11 (6.7) | 6 (8.6) | 5 (5.4) | 0.842 |
|  | 26-50% | 24 (14.7) | 11 (15.7) | 13 (14.0) |  |
|  | 51-75% | 42 (25.8) | 17 (24.3) | 25 (26.9) |  |
|  | 76-100% | 86 (52.8) | 36 (51.4) | 50 (53.8) |  |
| PR | 0-25% | 55 (33.7) | 25 (35.7) | 30 (32.3) | 0.324 |
|  | 26-50% | 29 (17.8) | 8 (11.4) | 21 (22.6) |  |
|  | 51-75% | 31 (19.0) | 15 (21.4) | 16 (17.2) |  |
|  | 76-100% | 48 (29.4) | 22 (31.4) | 26 (28.0) |  |
| HER2 | Negative | 113 (69.3) | 50 (71.4) | 63 (67.7) | 0.739 |
|  | Positive | 50 (30.7) | 20 (28.6) | 30 (32.3) |  |
| Ki67 | 0-25% | 113 (69.3) | 52 (74.3) | 61 (65.6) | 0.261 |
|  | 26-50% | 38 (23.3) | 12 (17.1) | 26 (28.0) |  |
|  | 51-75% | 12 (7.4) | 6 (8.6) | 6 (6.5) |  |
| CK5/6 | Negative | 130 (79.8) | 56 (80.0) | 74 (79.6) | 1.000 |
|  | Positive | 33 (20.2) | 14 (20.0) | 19 (20.4) |  |
| E-cad | Negative | 3 (1.8) | 1 (1.4) | 2 (2.2) | 1.000 |
|  | Positive | 160 (98.2) | 69 (98.6) | 91 (97.8) |  |
| P120 | Negative | 154 (94.5) | 64 (91.4) | 90 (96.8) | 0.257 |
|  | Positive | 9 (5.5) | 6 (8.6) | 3 (3.2) |  |
| P53 | Negative | 99 (60.7) | 51 (72.9) | 48 (51.6) | 0.010 |
|  | Positive | 64 (39.3) | 19 (27.1) | 45 (48.4) |  |
| Blood vessel invasion | No | 147 (90.2) | 65 (92.9) | 82 (88.2) | 0.466 |
|  | Yes | 16 (9.8) | 5 (7.1) | 11 (11.8) |  |
| Lung metastasis | No | 150 (92.0) | 66 (94.3) | 84 (90.3) | 0.527 |
|  | Yes | 13 (8.0) | 4 (5.7) | 9 (9.7) |  |
| Bone metastasis | No | 144 (88.3) | 59 (84.3) | 85 (91.4) | 0.248 |
|  | Yes | 19 (11.7) | 11 (15.7) | 8 (8.6) |  |
| Liver metastasis | No | 149 (91.4) | 60 (85.7) | 89 (95.7) | 0.049 |
|  | Yes | 14 (8.6) | 10 (14.3) | 4 (4.3) |  |
| Mediastinal metastasis | No | 158 (96.9) | 67 (95.7) | 91 (97.8) | 0.746 |
|  | Yes | 5 (3.1) | 3 (4.3) | 2 (2.2) |  |
| Brain metastasis | No | 159 (97.5) | 68 (97.1) | 91 (97.8) | 1.000 |
|  | Yes | 4 (2.5) | 2 (2.9) | 2 (2.2) |  |
| Pleural metastasis | No | 156 (95.7) | 66 (94.3) | 90 (96.8) | 0.700 |
|  | Yes | 7 (4.3) | 4 (5.7) | 3 (3.2) |  |
| Chest wall metastasis | No | 161 (98.8) | 69 (98.6) | 92 (98.9) | 1.000 |
|  | Yes | 2 (1.2) | 1 (1.4) | 1 (1.1) |  |
| Axillary metastasis | No | 80 (49.1) | 32 (45.7) | 48 (51.6) | 0.557 |
|  | Yes | 83 (50.9) | 38 (54.3) | 45 (48.4) |  |
| Clavicle metastasis | No | 145 (89.0) | 63 (90.0) | 82 (88.2) | 0.908 |
|  | Yes | 18 (11.0) | 7 (10.0) | 11 (11.8) |  |
| Chemotherapy | No | 14 (8.6) | 7 (10.0) | 7 (7.5) | 0.783 |
|  | Yes | 149 (91.4) | 63 (90.0) | 86 (92.5) |  |
| Radiotherapy | No | 101 (62.0) | 40 (57.1) | 61 (65.6) | 0.349 |
|  | Yes | 62 (38.0) | 30 (42.9) | 32 (34.4) |  |
| Endocrine therapy drugs | Anastrozole | 14 (8.6) | 10 (14.3) | 4 (4.3) | 0.017 |
|  | Letrozole | 42 (25.8) | 10 (14.3) | 32 (34.4) |  |
|  | Tamoxifen | 90 (55.2) | 42 (60.0) | 48 (51.6) |  |
|  | Toremifen | 8 (4.9) | 3 (4.3) | 5 (5.4) |  |
|  | Exemestane | 9 (5.5) | 5 (7.1) | 4 (4.3) |  |
| Targeted therapy | No | 153 (93.9) | 64 (91.4) | 89 (95.7) | 0.427 |
|  | Yes | 10 (6.1) | 6 (8.6) | 4 (4.3) |  |

**Table S3** Demographic and clinicopathologic characteristics of patients with breast cancer received radiotherapy

|  | level | Overall | Low TOX | High TOX | p |
| --- | --- | --- | --- | --- | --- |
| n |  | 93 | 43 | 50 |  |
| Age | <51 | 51 (54.8) | 23 (53.5) | 28 (56.0) | 0.973 |
|  | ≥51 | 42 (45.2) | 20 (46.5) | 22 (44.0) |  |
| Weight | <62 | 40 (43.0) | 18 (41.9) | 22 (44.0) | 1.000 |
|  | ≥62 | 53 (57.0) | 25 (58.1) | 28 (56.0) |  |
| Height | <1.60 | 30 (32.3) | 11 (25.6) | 19 (38.0) | 0.291 |
|  | ≥1.60 | 63 (67.7) | 32 (74.4) | 31 (62.0) |  |
| BMI | <23.8 | 48 (51.6) | 22 (51.2) | 26 (52.0) | 1.000 |
|  | ≥23.8 | 45 (48.4) | 21 (48.8) | 24 (48.0) |  |
| Family history | No | 75 (80.6) | 31 (72.1) | 44 (88.0) | 0.094 |
|  | Yes | 18 (19.4) | 12 (27.9) | 6 (12.0) |  |
| Basic disease | No | 74 (79.6) | 34 (79.1) | 40 (80.0) | 1.000 |
|  | Yes | 19 (20.4) | 9 (20.9) | 10 (20.0) |  |
| Hypertension | No | 83 (89.2) | 38 (88.4) | 45 (90.0) | 1.000 |
|  | Yes | 10 (10.8) | 5 (11.6) | 5 (10.0) |  |
| Diabetes mellitus | No | 86 (92.5) | 40 (93.0) | 46 (92.0) | 1.000 |
|  | Yes | 7 (7.5) | 3 (7.0) | 4 (8.0) |  |
| Coronary heart disease | No | 88 (94.6) | 41 (95.3) | 47 (94.0) | 1.000 |
|  | Yes | 5 (5.4) | 2 (4.7) | 3 (6.0) |  |
| Menarche age | <15 | 38 (40.9) | 17 (39.5) | 21 (42.0) | 0.976 |
|  | ≥15 | 55 (59.1) | 26 (60.5) | 29 (58.0) |  |
| Menopause | No | 48 (51.6) | 22 (51.2) | 26 (52.0) | 1.000 |
|  | Yes | 45 (48.4) | 21 (48.8) | 24 (48.0) |  |
| ALT | <21 | 39 (41.9) | 17 (39.5) | 22 (44.0) | 0.822 |
|  | ≥21 | 54 (58.1) | 26 (60.5) | 28 (56.0) |  |
| AST | <23 | 37 (39.8) | 15 (34.9) | 22 (44.0) | 0.495 |
|  | ≥23 | 56 (60.2) | 28 (65.1) | 28 (56.0) |  |
| AST/ALT | <1.1 | 46 (49.5) | 21 (48.8) | 25 (50.0) | 1.000 |
|  | ≥1.1 | 47 (50.5) | 22 (51.2) | 25 (50.0) |  |
| LDH | <170 | 46 (49.5) | 23 (53.5) | 23 (46.0) | 0.609 |
|  | ≥170 | 47 (50.5) | 20 (46.5) | 27 (54.0) |  |
| GGT | <14 | 50 (53.8) | 21 (48.8) | 29 (58.0) | 0.500 |
|  | ≥14 | 43 (46.2) | 22 (51.2) | 21 (42.0) |  |
| ALP | <70 | 48 (51.6) | 21 (48.8) | 27 (54.0) | 0.773 |
|  | ≥70 | 45 (48.4) | 22 (51.2) | 23 (46.0) |  |
| GLU | <5.1 | 43 (46.2) | 19 (44.2) | 24 (48.0) | 0.873 |
|  | ≥5.1 | 50 (53.8) | 24 (55.8) | 26 (52.0) |  |
| ALB | <45 | 45 (48.4) | 22 (51.2) | 23 (46.0) | 0.773 |
|  | ≥45 | 48 (51.6) | 21 (48.8) | 27 (54.0) |  |
| BUN | <4.9 | 50 (53.8) | 26 (60.5) | 24 (48.0) | 0.320 |
|  | ≥4.9 | 43 (46.2) | 17 (39.5) | 26 (52.0) |  |
| UR/CR | <0.078 | 52 (55.9) | 25 (58.1) | 27 (54.0) | 0.848 |
|  | ≥0.078 | 41 (44.1) | 18 (41.9) | 23 (46.0) |  |
| CRE | <63 | 52 (55.9) | 24 (55.8) | 28 (56.0) | 1.000 |
|  | ≥63 | 41 (44.1) | 19 (44.2) | 22 (44.0) |  |
| URIC | <253 | 41 (44.1) | 18 (41.9) | 23 (46.0) | 0.848 |
|  | ≥253 | 52 (55.9) | 25 (58.1) | 27 (54.0) |  |
| TBIL | <12.45 | 38 (40.9) | 17 (39.5) | 21 (42.0) | 0.976 |
|  | ≥12.45 | 55 (59.1) | 26 (60.5) | 29 (58.0) |  |
| DBIL | <3.9 | 35 (37.6) | 15 (34.9) | 20 (40.0) | 0.769 |
|  | ≥3.9 | 58 (62.4) | 28 (65.1) | 30 (60.0) |  |
| IBIL | <8.29 | 39 (41.9) | 16 (37.2) | 23 (46.0) | 0.518 |
|  | ≥8.29 | 54 (58.1) | 27 (62.8) | 27 (54.0) |  |
| TP | <74 | 38 (40.9) | 16 (37.2) | 22 (44.0) | 0.651 |
|  | ≥74 | 55 (59.1) | 27 (62.8) | 28 (56.0) |  |
| G | <29 | 36 (38.7) | 12 (27.9) | 24 (48.0) | 0.077 |
|  | ≥29 | 57 (61.3) | 31 (72.1) | 26 (52.0) |  |
| A/G | <1.5 | 35 (37.6) | 16 (37.2) | 19 (38.0) | 1.000 |
|  | ≥1.5 | 58 (62.4) | 27 (62.8) | 31 (62.0) |  |
| PAB | <267 | 48 (51.6) | 17 (39.5) | 31 (62.0) | 0.051 |
|  | ≥267 | 45 (48.4) | 26 (60.5) | 19 (38.0) |  |
| CO2 | <28.5 | 38 (40.9) | 16 (37.2) | 22 (44.0) | 0.651 |
|  | ≥28.5 | 55 (59.1) | 27 (62.8) | 28 (56.0) |  |
| CA153 | <9.82 | 41 (44.1) | 16 (37.2) | 25 (50.0) | 0.303 |
|  | ≥9.82 | 52 (55.9) | 27 (62.8) | 25 (50.0) |  |
| CEA | <1.49 | 45 (48.4) | 18 (41.9) | 27 (54.0) | 0.337 |
|  | ≥1.49 | 48 (51.6) | 25 (58.1) | 23 (46.0) |  |
| D-D | <0.25 | 47 (50.5) | 27 (62.8) | 20 (40.0) | 0.047 |
|  | ≥0.25 | 46 (49.5) | 16 (37.2) | 30 (60.0) |  |
| FBG | <2.6 | 47 (50.5) | 24 (55.8) | 23 (46.0) | 0.462 |
|  | ≥2.6 | 46 (49.5) | 19 (44.2) | 27 (54.0) |  |
| INR | <0.97 | 41 (44.1) | 22 (51.2) | 19 (38.0) | 0.287 |
|  | ≥0.97 | 52 (55.9) | 21 (48.8) | 31 (62.0) |  |
| PT | <11.1 | 41 (44.1) | 22 (51.2) | 19 (38.0) | 0.287 |
|  | ≥11.1 | 52 (55.9) | 21 (48.8) | 31 (62.0) |  |
| APTT | <27.5 | 46 (49.5) | 22 (51.2) | 24 (48.0) | 0.923 |
|  | ≥27.5 | 47 (50.5) | 21 (48.8) | 26 (52.0) |  |
| TT | <17.2 | 48 (51.6) | 19 (44.2) | 29 (58.0) | 0.262 |
|  | ≥17.2 | 45 (48.4) | 24 (55.8) | 21 (42.0) |  |
| Blood type | A | 19 (20.4) | 9 (20.9) | 10 (20.0) | 0.503 |
|  | B | 36 (38.7) | 15 (34.9) | 21 (42.0) |  |
|  | O | 24 (25.8) | 14 (32.6) | 10 (20.0) |  |
|  | AB | 14 (15.1) | 5 (11.6) | 9 (18.0) |  |
| White blood cell | <5.45 | 44 (47.3) | 20 (46.5) | 24 (48.0) | 1.000 |
|  | ≥5.45 | 49 (52.7) | 23 (53.5) | 26 (52.0) |  |
| Red blood cell | <4.2 | 45 (48.4) | 19 (44.2) | 26 (52.0) | 0.587 |
|  | ≥4.2 | 48 (51.6) | 24 (55.8) | 24 (48.0) |  |
| Hemoglobin | <127 | 42 (45.2) | 17 (39.5) | 25 (50.0) | 0.422 |
|  | ≥127 | 51 (54.8) | 26 (60.5) | 25 (50.0) |  |
| Neutrophil | <3.23 | 48 (51.6) | 24 (55.8) | 24 (48.0) | 0.587 |
|  | ≥3.23 | 45 (48.4) | 19 (44.2) | 26 (52.0) |  |
| Lymphocyte | <1.70 | 45 (48.4) | 20 (46.5) | 25 (50.0) | 0.899 |
|  | ≥1.70 | 48 (51.6) | 23 (53.5) | 25 (50.0) |  |
| Monocyte | <0.35 | 47 (50.5) | 19 (44.2) | 28 (56.0) | 0.353 |
|  | ≥0.35 | 46 (49.5) | 24 (55.8) | 22 (44.0) |  |
| Eosinophil | <0.07 | 53 (57.0) | 28 (65.1) | 25 (50.0) | 0.208 |
|  | ≥0.07 | 40 (43.0) | 15 (34.9) | 25 (50.0) |  |
| Basophil | <0.02 | 45 (48.4) | 19 (44.2) | 26 (52.0) | 0.587 |
|  | ≥0.02 | 48 (51.6) | 24 (55.8) | 24 (48.0) |  |
| Platelet | <233 | 46 (49.5) | 17 (39.5) | 29 (58.0) | 0.117 |
|  | ≥233 | 47 (50.5) | 26 (60.5) | 21 (42.0) |  |
| Primary tumor site | Upper outer quadrant | 53 (57.0) | 25 (58.1) | 28 (56.0) | 0.835 |
|  | Lower outer quadrant | 9 (9.7) | 4 (9.3) | 5 (10.0) |  |
|  | Lower inner quadrant | 6 (6.5) | 4 (9.3) | 2 (4.0) |  |
|  | Upper inner quadrant | 13 (14.0) | 5 (11.6) | 8 (16.0) |  |
|  | Central | 12 (12.9) | 5 (11.6) | 7 (14.0) |  |
| US-BIRADS | BIRADS 4 | 39 (41.9) | 18 (41.9) | 21 (42.0) | 0.994 |
|  | BIRADS 5 | 52 (55.9) | 24 (55.8) | 28 (56.0) |  |
|  | BIRADS 6 | 2 (2.2) | 1 (2.3) | 1 (2.0) |  |
| Operative time | <75 | 37 (39.8) | 13 (30.2) | 24 (48.0) | 0.125 |
|  | ≥75 | 56 (60.2) | 30 (69.8) | 26 (52.0) |  |
| Type of surgery | Mastectomy | 77 (82.8) | 35 (81.4) | 42 (84.0) | 0.955 |
|  | Breast-conserving surgery | 16 (17.2) | 8 (18.6) | 8 (16.0) |  |
| Tumor size | ≤2 | 45 (48.4) | 20 (46.5) | 25 (50.0) | 0.942 |
|  | ＞2 and＜5 | 44 (47.3) | 21 (48.8) | 23 (46.0) |  |
|  | ≥5 | 4 (4.3) | 2 (4.7) | 2 (4.0) |  |
| Histologic grade | I | 1 (1.1) | 0 (0.0) | 1 (2.0) | 0.443 |
|  | II | 56 (60.2) | 27 (62.8) | 29 (58.0) |  |
|  | III | 32 (34.4) | 13 (30.2) | 19 (38.0) |  |
|  | Unknown | 4 (4.3) | 3 (7.0) | 1 (2.0) |  |
| Pathological T Stage | T1 | 45 (48.4) | 19 (44.2) | 26 (52.0) | 0.852 |
|  | T2 | 41 (44.1) | 21 (48.8) | 20 (40.0) |  |
|  | T3 | 5 (5.4) | 2 (4.7) | 3 (6.0) |  |
|  | T4 | 2 (2.2) | 1 (2.3) | 1 (2.0) |  |
| Pathological N Stage | N0 | 16 (17.2) | 7 (16.3) | 9 (18.0) | 0.849 |
|  | N1 | 23 (24.7) | 10 (23.3) | 13 (26.0) |  |
|  | N2 | 32 (34.4) | 14 (32.6) | 18 (36.0) |  |
|  | N3 | 22 (23.7) | 12 (27.9) | 10 (20.0) |  |
| Pathological TNM Stage | I | 12 (12.9) | 6 (14.0) | 6 (12.0) | 0.760 |
|  | II | 25 (26.9) | 10 (23.3) | 15 (30.0) |  |
|  | III | 56 (60.2) | 27 (62.8) | 29 (58.0) |  |
| TLN | <16 | 37 (39.8) | 18 (41.9) | 19 (38.0) | 0.868 |
|  | ≥16 | 56 (60.2) | 25 (58.1) | 31 (62.0) |  |
| PLN | <1 | 17 (18.3) | 8 (18.6) | 9 (18.0) | 1.000 |
|  | ≥1 | 76 (81.7) | 35 (81.4) | 41 (82.0) |  |
| TALN | <14 | 38 (40.9) | 21 (48.8) | 17 (34.0) | 0.215 |
|  | ≥14 | 55 (59.1) | 22 (51.2) | 33 (66.0) |  |
| PALN | <1 | 25 (26.9) | 13 (30.2) | 12 (24.0) | 0.659 |
|  | ≥1 | 68 (73.1) | 30 (69.8) | 38 (76.0) |  |
| Molecular subtype | Luminal A | 19 (20.4) | 11 (25.6) | 8 (16.0) | 0.852 |
|  | Luminal B HER2+ | 20 (21.5) | 9 (20.9) | 11 (22.0) |  |
|  | Luminal B HER2- | 24 (25.8) | 10 (23.3) | 14 (28.0) |  |
|  | HER2 enriched | 14 (15.1) | 6 (14.0) | 8 (16.0) |  |
|  | Triple negative | 16 (17.2) | 7 (16.3) | 9 (18.0) |  |
| ER | 0-25% | 36 (38.7) | 18 (41.9) | 18 (36.0) | 0.675 |
|  | 26-50% | 11 (11.8) | 6 (14.0) | 5 (10.0) |  |
|  | 51-75% | 15 (16.1) | 5 (11.6) | 10 (20.0) |  |
|  | 76-100% | 31 (33.3) | 14 (32.6) | 17 (34.0) |  |
| PR | 0-25% | 57 (61.3) | 27 (62.8) | 30 (60.0) | 0.809 |
|  | 26-50% | 6 (6.5) | 2 (4.7) | 4 (8.0) |  |
|  | 51-75% | 15 (16.1) | 6 (14.0) | 9 (18.0) |  |
|  | 76-100% | 15 (16.1) | 8 (18.6) | 7 (14.0) |  |
| HER2 | Negative | 59 (63.4) | 28 (65.1) | 31 (62.0) | 0.924 |
|  | Positive | 34 (36.6) | 15 (34.9) | 19 (38.0) |  |
| Ki67 | 0-25% | 49 (52.7) | 28 (65.1) | 21 (42.0) | 0.053 |
|  | 26-50% | 26 (28.0) | 11 (25.6) | 15 (30.0) |  |
|  | 51-75% | 14 (15.1) | 4 (9.3) | 10 (20.0) |  |
|  | 76-100% | 4 (4.3) | 0 (0.0) | 4 (8.0) |  |
| CK5/6 | Negative | 65 (69.9) | 31 (72.1) | 34 (68.0) | 0.840 |
|  | Positive | 28 (30.1) | 12 (27.9) | 16 (32.0) |  |
| E-cad | Negative | 3 (3.2) | 2 (4.7) | 1 (2.0) | 0.894 |
|  | Positive | 90 (96.8) | 41 (95.3) | 49 (98.0) |  |
| P120 | Negative | 84 (90.3) | 36 (83.7) | 48 (96.0) | 0.100 |
|  | Positive | 9 (9.7) | 7 (16.3) | 2 (4.0) |  |
| P53 | Negative | 51 (54.8) | 25 (58.1) | 26 (52.0) | 0.701 |
|  | Positive | 42 (45.2) | 18 (41.9) | 24 (48.0) |  |
| Blood vessel invasion | No | 81 (87.1) | 37 (86.0) | 44 (88.0) | 1.000 |
|  | Yes | 12 (12.9) | 6 (14.0) | 6 (12.0) |  |
| Lung metastasis | No | 78 (83.9) | 38 (88.4) | 40 (80.0) | 0.417 |
|  | Yes | 15 (16.1) | 5 (11.6) | 10 (20.0) |  |
| Bone metastasis | No | 74 (79.6) | 34 (79.1) | 40 (80.0) | 1.000 |
|  | Yes | 19 (20.4) | 9 (20.9) | 10 (20.0) |  |
| Liver metastasis | No | 79 (84.9) | 35 (81.4) | 44 (88.0) | 0.550 |
|  | Yes | 14 (15.1) | 8 (18.6) | 6 (12.0) |  |
| Mediastinal metastasis | No | 87 (93.5) | 39 (90.7) | 48 (96.0) | 0.539 |
|  | Yes | 6 (6.5) | 4 (9.3) | 2 (4.0) |  |
| Brain metastasis | No | 85 (91.4) | 38 (88.4) | 47 (94.0) | 0.552 |
|  | Yes | 8 (8.6) | 5 (11.6) | 3 (6.0) |  |
| Pleural metastasis | No | 87 (93.5) | 39 (90.7) | 48 (96.0) | 0.539 |
|  | Yes | 6 (6.5) | 4 (9.3) | 2 (4.0) |  |
| Chest wall metastasis | No | 90 (96.8) | 41 (95.3) | 49 (98.0) | 0.894 |
|  | Yes | 3 (3.2) | 2 (4.7) | 1 (2.0) |  |
| Axillary metastasis | No | 25 (26.9) | 13 (30.2) | 12 (24.0) | 0.659 |
|  | Yes | 68 (73.1) | 30 (69.8) | 38 (76.0) |  |
| Clavicle metastasis | No | 73 (78.5) | 35 (81.4) | 38 (76.0) | 0.705 |
|  | Yes | 20 (21.5) | 8 (18.6) | 12 (24.0) |  |
| Chemotherapy | No | 3 (3.2) | 2 (4.7) | 1 (2.0) | 0.894 |
|  | Yes | 90 (96.8) | 41 (95.3) | 49 (98.0) |  |
| Chemotherapy times | <7 | 36 (38.7) | 19 (44.2) | 17 (34.0) | 0.428 |
|  | ≥7 | 57 (61.3) | 24 (55.8) | 33 (66.0) |  |
| Endocrine therapy | No | 31 (33.3) | 13 (30.2) | 18 (36.0) | 0.713 |
|  | Yes | 62 (66.7) | 30 (69.8) | 32 (64.0) |  |
| Targeted therapy | No | 77 (82.8) | 35 (81.4) | 42 (84.0) | 0.955 |
|  | Yes | 16 (17.2) | 8 (18.6) | 8 (16.0) |  |

**Table S4** Demographic and clinicopathologic characteristics of breast cancer patients with stage I

|  | level | Overall | Low TOX | High TOX | p |
| --- | --- | --- | --- | --- | --- |
| n |  | 85 | 36 | 49 |  |
| Age | <51 | 41 (48.2) | 19 (52.8) | 22 (44.9) | 0.618 |
|  | ≥51 | 44 (51.8) | 17 (47.2) | 27 (55.1) |  |
| Weight | <62 | 45 (52.9) | 22 (61.1) | 23 (46.9) | 0.283 |
|  | ≥62 | 40 (47.1) | 14 (38.9) | 26 (53.1) |  |
| Height | <1.60 | 26 (30.6) | 14 (38.9) | 12 (24.5) | 0.236 |
|  | ≥1.60 | 59 (69.4) | 22 (61.1) | 37 (75.5) |  |
| BMI | <23.8 | 44 (51.8) | 21 (58.3) | 23 (46.9) | 0.413 |
|  | ≥23.8 | 41 (48.2) | 15 (41.7) | 26 (53.1) |  |
| Family history | No | 55 (64.7) | 24 (66.7) | 31 (63.3) | 0.925 |
|  | Yes | 30 (35.3) | 12 (33.3) | 18 (36.7) |  |
| Basic disease | No | 65 (76.5) | 27 (75.0) | 38 (77.6) | 0.988 |
|  | Yes | 20 (23.5) | 9 (25.0) | 11 (22.4) |  |
| Hypertension | No | 74 (87.1) | 31 (86.1) | 43 (87.8) | 1.000 |
|  | Yes | 11 (12.9) | 5 (13.9) | 6 (12.2) |  |
| Diabetes mellitus | No | 80 (94.1) | 35 (97.2) | 45 (91.8) | 0.564 |
|  | Yes | 5 (5.9) | 1 (2.8) | 4 (8.2) |  |
| Coronary heart disease | No | 81 (95.3) | 33 (91.7) | 48 (98.0) | 0.404 |
|  | Yes | 4 (4.7) | 3 (8.3) | 1 (2.0) |  |
| Menarche age | <15 | 33 (38.8) | 14 (38.9) | 19 (38.8) | 1.000 |
|  | ≥15 | 52 (61.2) | 22 (61.1) | 30 (61.2) |  |
| Menopause | No | 43 (50.6) | 17 (47.2) | 26 (53.1) | 0.755 |
|  | Yes | 42 (49.4) | 19 (52.8) | 23 (46.9) |  |
| ALT | <21 | 44 (51.8) | 17 (47.2) | 27 (55.1) | 0.618 |
|  | ≥21 | 41 (48.2) | 19 (52.8) | 22 (44.9) |  |
| AST | <23 | 41 (48.2) | 14 (38.9) | 27 (55.1) | 0.208 |
|  | ≥23 | 44 (51.8) | 22 (61.1) | 22 (44.9) |  |
| AST/ALT | <1.1 | 38 (44.7) | 17 (47.2) | 21 (42.9) | 0.858 |
|  | ≥1.1 | 47 (55.3) | 19 (52.8) | 28 (57.1) |  |
| LDH | <170 | 52 (61.2) | 18 (50.0) | 34 (69.4) | 0.112 |
|  | ≥170 | 33 (38.8) | 18 (50.0) | 15 (30.6) |  |
| GGT | <14 | 39 (45.9) | 14 (38.9) | 25 (51.0) | 0.374 |
|  | ≥14 | 46 (54.1) | 22 (61.1) | 24 (49.0) |  |
| ALP | <70 | 45 (52.9) | 20 (55.6) | 25 (51.0) | 0.846 |
|  | ≥70 | 40 (47.1) | 16 (44.4) | 24 (49.0) |  |
| GLU | <5.1 | 38 (44.7) | 15 (41.7) | 23 (46.9) | 0.793 |
|  | ≥5.1 | 47 (55.3) | 21 (58.3) | 26 (53.1) |  |
| ALB | <45 | 41 (48.2) | 13 (36.1) | 28 (57.1) | 0.090 |
|  | ≥45 | 44 (51.8) | 23 (63.9) | 21 (42.9) |  |
| BUN | <4.9 | 44 (51.8) | 25 (69.4) | 19 (38.8) | 0.010 |
|  | ≥4.9 | 41 (48.2) | 11 (30.6) | 30 (61.2) |  |
| UR/CR | <0.078 | 43 (50.6) | 22 (61.1) | 21 (42.9) | 0.149 |
|  | ≥0.078 | 42 (49.4) | 14 (38.9) | 28 (57.1) |  |
| CRE | <63 | 39 (45.9) | 18 (50.0) | 21 (42.9) | 0.665 |
|  | ≥63 | 46 (54.1) | 18 (50.0) | 28 (57.1) |  |
| URIC | <253 | 44 (51.8) | 17 (47.2) | 27 (55.1) | 0.618 |
|  | ≥253 | 41 (48.2) | 19 (52.8) | 22 (44.9) |  |
| TBIL | <12.45 | 55 (64.7) | 21 (58.3) | 34 (69.4) | 0.410 |
|  | ≥12.45 | 30 (35.3) | 15 (41.7) | 15 (30.6) |  |
| DBIL | <3.9 | 58 (68.2) | 24 (66.7) | 34 (69.4) | 0.976 |
|  | ≥3.9 | 27 (31.8) | 12 (33.3) | 15 (30.6) |  |
| IBIL | <8.29 | 53 (62.4) | 21 (58.3) | 32 (65.3) | 0.668 |
|  | ≥8.29 | 32 (37.6) | 15 (41.7) | 17 (34.7) |  |
| TP | <74 | 38 (44.7) | 13 (36.1) | 25 (51.0) | 0.252 |
|  | ≥74 | 47 (55.3) | 23 (63.9) | 24 (49.0) |  |
| G | <29 | 42 (49.4) | 17 (47.2) | 25 (51.0) | 0.899 |
|  | ≥29 | 43 (50.6) | 19 (52.8) | 24 (49.0) |  |
| A/G | <1.5 | 25 (29.4) | 8 (22.2) | 17 (34.7) | 0.314 |
|  | ≥1.5 | 60 (70.6) | 28 (77.8) | 32 (65.3) |  |
| PAB | <267 | 36 (42.4) | 16 (44.4) | 20 (40.8) | 0.911 |
|  | ≥267 | 49 (57.6) | 20 (55.6) | 29 (59.2) |  |
| CO2 | <28.5 | 40 (47.1) | 19 (52.8) | 21 (42.9) | 0.493 |
|  | ≥28.5 | 45 (52.9) | 17 (47.2) | 28 (57.1) |  |
| CA153 | <9.82 | 52 (61.2) | 17 (47.2) | 35 (71.4) | 0.042 |
|  | ≥9.82 | 33 (38.8) | 19 (52.8) | 14 (28.6) |  |
| CEA | <1.49 | 50 (58.8) | 23 (63.9) | 27 (55.1) | 0.555 |
|  | ≥1.49 | 35 (41.2) | 13 (36.1) | 22 (44.9) |  |
| D-D | <0.25 | 42 (49.4) | 19 (52.8) | 23 (46.9) | 0.755 |
|  | ≥0.25 | 43 (50.6) | 17 (47.2) | 26 (53.1) |  |
| FBG | <2.6 | 39 (45.9) | 15 (41.7) | 24 (49.0) | 0.654 |
|  | ≥2.6 | 46 (54.1) | 21 (58.3) | 25 (51.0) |  |
| INR | <0.97 | 38 (44.7) | 15 (41.7) | 23 (46.9) | 0.793 |
|  | ≥0.97 | 47 (55.3) | 21 (58.3) | 26 (53.1) |  |
| PT | <11.1 | 38 (44.7) | 15 (41.7) | 23 (46.9) | 0.793 |
|  | ≥11.1 | 47 (55.3) | 21 (58.3) | 26 (53.1) |  |
| APTT | <27.5 | 40 (47.1) | 18 (50.0) | 22 (44.9) | 0.806 |
|  | ≥27.5 | 45 (52.9) | 18 (50.0) | 27 (55.1) |  |
| TT | <17.2 | 46 (54.1) | 19 (52.8) | 27 (55.1) | 1.000 |
|  | ≥17.2 | 39 (45.9) | 17 (47.2) | 22 (44.9) |  |
| Blood type | A | 20 (23.5) | 5 (13.9) | 15 (30.6) | 0.275 |
|  | B | 32 (37.6) | 14 (38.9) | 18 (36.7) |  |
|  | O | 22 (25.9) | 12 (33.3) | 10 (20.4) |  |
|  | AB | 11 (12.9) | 5 (13.9) | 6 (12.2) |  |
| White blood cell | <5.45 | 50 (58.8) | 26 (72.2) | 24 (49.0) | 0.054 |
|  | ≥5.45 | 35 (41.2) | 10 (27.8) | 25 (51.0) |  |
| Red blood cell | <4.2 | 44 (51.8) | 19 (52.8) | 25 (51.0) | 1.000 |
|  | ≥4.2 | 41 (48.2) | 17 (47.2) | 24 (49.0) |  |
| Hemoglobin | <127 | 46 (54.1) | 20 (55.6) | 26 (53.1) | 0.994 |
|  | ≥127 | 39 (45.9) | 16 (44.4) | 23 (46.9) |  |
| Neutrophil | <3.23 | 47 (55.3) | 21 (58.3) | 26 (53.1) | 0.793 |
|  | ≥3.23 | 38 (44.7) | 15 (41.7) | 23 (46.9) |  |
| Lymphocyte | <1.70 | 42 (49.4) | 17 (47.2) | 25 (51.0) | 0.899 |
|  | ≥1.70 | 43 (50.6) | 19 (52.8) | 24 (49.0) |  |
| Monocyte | <0.35 | 47 (55.3) | 22 (61.1) | 25 (51.0) | 0.482 |
|  | ≥0.35 | 38 (44.7) | 14 (38.9) | 24 (49.0) |  |
| Eosinophil | <0.07 | 42 (49.4) | 17 (47.2) | 25 (51.0) | 0.899 |
|  | ≥0.07 | 43 (50.6) | 19 (52.8) | 24 (49.0) |  |
| Basophil | <0.02 | 38 (44.7) | 17 (47.2) | 21 (42.9) | 0.858 |
|  | ≥0.02 | 47 (55.3) | 19 (52.8) | 28 (57.1) |  |
| Platelet | <233 | 40 (47.1) | 17 (47.2) | 23 (46.9) | 1.000 |
|  | ≥233 | 45 (52.9) | 19 (52.8) | 26 (53.1) |  |
| Tumor site | Right | 37 (43.5) | 11 (30.6) | 26 (53.1) | 0.065 |
|  | Left | 48 (56.5) | 25 (69.4) | 23 (46.9) |  |
| Primary tumor site | Upper outer quadrant | 45 (52.9) | 17 (47.2) | 28 (57.1) | 0.258 |
|  | Lower outer quadrant | 6 (7.1) | 2 (5.6) | 4 (8.2) |  |
|  | Lower inner quadrant | 8 (9.4) | 3 (8.3) | 5 (10.2) |  |
|  | Upper inner quadrant | 17 (20.0) | 7 (19.4) | 10 (20.4) |  |
|  | Central | 9 (10.6) | 7 (19.4) | 2 (4.1) |  |
| US-BIRADS | BIRADS 4 | 60 (70.6) | 25 (69.4) | 35 (71.4) | 0.925 |
|  | BIRADS 5 | 19 (22.4) | 8 (22.2) | 11 (22.4) |  |
|  | BIRADS 6 | 6 (7.1) | 3 (8.3) | 3 (6.1) |  |
| Operative time | <75 | 42 (49.4) | 16 (44.4) | 26 (53.1) | 0.572 |
|  | ≥75 | 43 (50.6) | 20 (55.6) | 23 (46.9) |  |
| Type of surgery | Mastectomy | 71 (83.5) | 28 (77.8) | 43 (87.8) | 0.353 |
|  | Breast-conserving surgery | 14 (16.5) | 8 (22.2) | 6 (12.2) |  |
| Histologic grade | I | 4 (4.7) | 1 (2.8) | 3 (6.1) | 0.866 |
|  | II | 56 (65.9) | 25 (69.4) | 31 (63.3) |  |
|  | III | 22 (25.9) | 9 (25.0) | 13 (26.5) |  |
|  | Unknown | 3 (3.5) | 1 (2.8) | 2 (4.1) |  |
| Molecular subtype | Luminal A | 24 (28.2) | 11 (30.6) | 13 (26.5) | 0.969 |
|  | Luminal B HER2+ | 18 (21.2) | 7 (19.4) | 11 (22.4) |  |
|  | Luminal B HER2- | 7 (8.2) | 3 (8.3) | 4 (8.2) |  |
|  | HER2 enriched | 14 (16.5) | 5 (13.9) | 9 (18.4) |  |
|  | Triple negative | 22 (25.9) | 10 (27.8) | 12 (24.5) |  |
| ER | 0-25% | 38 (44.7) | 16 (44.4) | 22 (44.9) | 0.848 |
|  | 26-50% | 9 (10.6) | 5 (13.9) | 4 (8.2) |  |
|  | 51-75% | 12 (14.1) | 5 (13.9) | 7 (14.3) |  |
|  | 76-100% | 26 (30.6) | 10 (27.8) | 16 (32.7) |  |
| PR | 0-25% | 51 (60.0) | 22 (61.1) | 29 (59.2) | 0.641 |
|  | 26-50% | 11 (12.9) | 3 (8.3) | 8 (16.3) |  |
|  | 51-75% | 9 (10.6) | 5 (13.9) | 4 (8.2) |  |
|  | 76-100% | 14 (16.5) | 6 (16.7) | 8 (16.3) |  |
| HER2 | Negative | 53 (62.4) | 24 (66.7) | 29 (59.2) | 0.633 |
|  | Positive | 32 (37.6) | 12 (33.3) | 20 (40.8) |  |
| Ki67 | 0-25% | 45 (52.9) | 21 (58.3) | 24 (49.0) | 0.284 |
|  | 26-50% | 24 (28.2) | 9 (25.0) | 15 (30.6) |  |
|  | 51-75% | 12 (14.1) | 3 (8.3) | 9 (18.4) |  |
|  | 76-100% | 4 (4.7) | 3 (8.3) | 1 (2.0) |  |
| CK5/6 | Negative | 58 (68.2) | 26 (72.2) | 32 (65.3) | 0.659 |
|  | Positive | 27 (31.8) | 10 (27.8) | 17 (34.7) |  |
| E-cad | Negative | 4 (4.7) | 4 (11.1) | 0 (0.0) | 0.061 |
|  | Positive | 81 (95.3) | 32 (88.9) | 49 (100.0) |  |
| P120 | Negative | 83 (97.6) | 35 (97.2) | 48 (98.0) | 1.000 |
|  | Positive | 2 (2.4) | 1 (2.8) | 1 (2.0) |  |
| P53 | Negative | 47 (55.3) | 20 (55.6) | 27 (55.1) | 1.000 |
|  | Positive | 38 (44.7) | 16 (44.4) | 22 (44.9) |  |
| Blood vessel invasion | No | 80 (94.1) | 35 (97.2) | 45 (91.8) | 0.564 |
|  | Yes | 5 (5.9) | 1 (2.8) | 4 (8.2) |  |
| Lung metastasis | No | 79 (92.9) | 34 (94.4) | 45 (91.8) | 0.972 |
|  | Yes | 6 (7.1) | 2 (5.6) | 4 (8.2) |  |
| Bone metastasis | No | 82 (96.5) | 35 (97.2) | 47 (95.9) | 1.000 |
|  | Yes | 3 (3.5) | 1 (2.8) | 2 (4.1) |  |
| Liver metastasis | No | 81 (95.3) | 32 (88.9) | 49 (100.0) | 0.061 |
|  | Yes | 4 (4.7) | 4 (11.1) | 0 (0.0) |  |
| Mediastinal metastasis | No | 82 (96.5) | 34 (94.4) | 48 (98.0) | 0.785 |
|  | Yes | 3 (3.5) | 2 (5.6) | 1 (2.0) |  |
| Brain metastasis | No | 82 (96.5) | 34 (94.4) | 48 (98.0) | 0.785 |
|  | Yes | 3 (3.5) | 2 (5.6) | 1 (2.0) |  |
| Pleural metastasis | No | 83 (97.6) | 36 (100.0) | 47 (95.9) | 0.615 |
|  | Yes | 2 (2.4) | 0 (0.0) | 2 (4.1) |  |
| Chest wall metastasis | No | 84 (98.8) | 36 (100.0) | 48 (98.0) | 1.000 |
|  | Yes | 1 (1.2) | 0 (0.0) | 1 (2.0) |  |
| Axillary metastasis | No | 80 (94.1) | 35 (97.2) | 45 (91.8) | 0.564 |
|  | Yes | 5 (5.9) | 1 (2.8) | 4 (8.2) |  |
| Clavicle metastasis | No | 77 (90.6) | 33 (91.7) | 44 (89.8) | 1.000 |
|  | Yes | 8 (9.4) | 3 (8.3) | 5 (10.2) |  |
| Chemotherapy | No | 15 (17.6) | 8 (22.2) | 7 (14.3) | 0.509 |
|  | Yes | 70 (82.4) | 28 (77.8) | 42 (85.7) |  |
| Radiotherapy | No | 73 (85.9) | 30 (83.3) | 43 (87.8) | 0.792 |
|  | Yes | 12 (14.1) | 6 (16.7) | 6 (12.2) |  |
| Endocrine therapy | No | 42 (49.4) | 19 (52.8) | 23 (46.9) | 0.755 |
|  | Yes | 43 (50.6) | 17 (47.2) | 26 (53.1) |  |
| Targeted therapy | No | 78 (91.8) | 32 (88.9) | 46 (93.9) | 0.669 |
|  | Yes | 7 (8.2) | 4 (11.1) | 3 (6.1) |  |

**Table S5** Demographic and clinicopathologic characteristics of breast cancer patients with stage II

|  | level | Overall | Low TOX | High TOX | p |
| --- | --- | --- | --- | --- | --- |
| n |  | 138 | 50 | 88 |  |
| Age | <51 | 72 (52.2) | 31 (62.0) | 41 (46.6) | 0.118 |
|  | ≥51 | 66 (47.8) | 19 (38.0) | 47 (53.4) |  |
| Weight | <62 | 63 (45.7) | 24 (48.0) | 39 (44.3) | 0.811 |
|  | ≥62 | 75 (54.3) | 26 (52.0) | 49 (55.7) |  |
| Height | <1.60 | 46 (33.3) | 17 (34.0) | 29 (33.0) | 1.000 |
|  | ≥1.60 | 92 (66.7) | 33 (66.0) | 59 (67.0) |  |
| BMI | <23.8 | 68 (49.3) | 25 (50.0) | 43 (48.9) | 1.000 |
|  | ≥23.8 | 70 (50.7) | 25 (50.0) | 45 (51.1) |  |
| Family history | No | 115 (83.3) | 42 (84.0) | 73 (83.0) | 1.000 |
|  | Yes | 23 (16.7) | 8 (16.0) | 15 (17.0) |  |
| Basic disease | No | 108 (78.3) | 35 (70.0) | 73 (83.0) | 0.119 |
|  | Yes | 30 (21.7) | 15 (30.0) | 15 (17.0) |  |
| Hypertension | No | 119 (86.2) | 39 (78.0) | 80 (90.9) | 0.063 |
|  | Yes | 19 (13.8) | 11 (22.0) | 8 (9.1) |  |
| Diabetes mellitus | No | 133 (96.4) | 47 (94.0) | 86 (97.7) | 0.514 |
|  | Yes | 5 (3.6) | 3 (6.0) | 2 (2.3) |  |
| Coronary heart disease | No | 133 (96.4) | 49 (98.0) | 84 (95.5) | 0.768 |
|  | Yes | 5 (3.6) | 1 (2.0) | 4 (4.5) |  |
| Menarche age | <15 | 55 (39.9) | 19 (38.0) | 36 (40.9) | 0.877 |
|  | ≥15 | 83 (60.1) | 31 (62.0) | 52 (59.1) |  |
| Menopause | No | 71 (51.4) | 32 (64.0) | 39 (44.3) | 0.041 |
|  | Yes | 67 (48.6) | 18 (36.0) | 49 (55.7) |  |
| ALT | <21 | 60 (43.5) | 23 (46.0) | 37 (42.0) | 0.786 |
|  | ≥21 | 78 (56.5) | 27 (54.0) | 51 (58.0) |  |
| AST | <23 | 67 (48.6) | 23 (46.0) | 44 (50.0) | 0.784 |
|  | ≥23 | 71 (51.4) | 27 (54.0) | 44 (50.0) |  |
| AST/ALT | <1.1 | 70 (50.7) | 25 (50.0) | 45 (51.1) | 1.000 |
|  | ≥1.1 | 68 (49.3) | 25 (50.0) | 43 (48.9) |  |
| LDH | <170 | 63 (45.7) | 25 (50.0) | 38 (43.2) | 0.552 |
|  | ≥170 | 75 (54.3) | 25 (50.0) | 50 (56.8) |  |
| GGT | <14 | 62 (44.9) | 22 (44.0) | 40 (45.5) | 1.000 |
|  | ≥14 | 76 (55.1) | 28 (56.0) | 48 (54.5) |  |
| ALP | <70 | 70 (50.7) | 26 (52.0) | 44 (50.0) | 0.961 |
|  | ≥70 | 68 (49.3) | 24 (48.0) | 44 (50.0) |  |
| GLU | <5.1 | 62 (44.9) | 25 (50.0) | 37 (42.0) | 0.468 |
|  | ≥5.1 | 76 (55.1) | 25 (50.0) | 51 (58.0) |  |
| ALB | <45 | 63 (45.7) | 23 (46.0) | 40 (45.5) | 1.000 |
|  | ≥45 | 75 (54.3) | 27 (54.0) | 48 (54.5) |  |
| BUN | <4.9 | 66 (47.8) | 25 (50.0) | 41 (46.6) | 0.835 |
|  | ≥4.9 | 72 (52.2) | 25 (50.0) | 47 (53.4) |  |
| UR/CR | <0.078 | 67 (48.6) | 25 (50.0) | 42 (47.7) | 0.937 |
|  | ≥0.078 | 71 (51.4) | 25 (50.0) | 46 (52.3) |  |
| CRE | <63 | 68 (49.3) | 25 (50.0) | 43 (48.9) | 1.000 |
|  | ≥63 | 70 (50.7) | 25 (50.0) | 45 (51.1) |  |
| URIC | <253 | 78 (56.5) | 27 (54.0) | 51 (58.0) | 0.786 |
|  | ≥253 | 60 (43.5) | 23 (46.0) | 37 (42.0) |  |
| TBIL | <12.45 | 66 (47.8) | 22 (44.0) | 44 (50.0) | 0.616 |
|  | ≥12.45 | 72 (52.2) | 28 (56.0) | 44 (50.0) |  |
| DBIL | <3.9 | 64 (46.4) | 18 (36.0) | 46 (52.3) | 0.096 |
|  | ≥3.9 | 74 (53.6) | 32 (64.0) | 42 (47.7) |  |
| IBIL | <8.29 | 65 (47.1) | 21 (42.0) | 44 (50.0) | 0.467 |
|  | ≥8.29 | 73 (52.9) | 29 (58.0) | 44 (50.0) |  |
| TP | <74 | 62 (44.9) | 24 (48.0) | 38 (43.2) | 0.712 |
|  | ≥74 | 76 (55.1) | 26 (52.0) | 50 (56.8) |  |
| G | <29 | 64 (46.4) | 23 (46.0) | 41 (46.6) | 1.000 |
|  | ≥29 | 74 (53.6) | 27 (54.0) | 47 (53.4) |  |
| A/G | <1.5 | 42 (30.4) | 11 (22.0) | 31 (35.2) | 0.152 |
|  | ≥1.5 | 96 (69.6) | 39 (78.0) | 57 (64.8) |  |
| PAB | <267 | 74 (53.6) | 24 (48.0) | 50 (56.8) | 0.412 |
|  | ≥267 | 64 (46.4) | 26 (52.0) | 38 (43.2) |  |
| CO2 | <28.5 | 78 (56.5) | 28 (56.0) | 50 (56.8) | 1.000 |
|  | ≥28.5 | 60 (43.5) | 22 (44.0) | 38 (43.2) |  |
| CA153 | <9.82 | 74 (53.6) | 24 (48.0) | 50 (56.8) | 0.412 |
|  | ≥9.82 | 64 (46.4) | 26 (52.0) | 38 (43.2) |  |
| CEA | <1.49 | 74 (53.6) | 29 (58.0) | 45 (51.1) | 0.549 |
|  | ≥1.49 | 64 (46.4) | 21 (42.0) | 43 (48.9) |  |
| D-D | <0.25 | 69 (50.0) | 29 (58.0) | 40 (45.5) | 0.215 |
|  | ≥0.25 | 69 (50.0) | 21 (42.0) | 48 (54.5) |  |
| FBG | <2.6 | 69 (50.0) | 28 (56.0) | 41 (46.6) | 0.376 |
|  | ≥2.6 | 69 (50.0) | 22 (44.0) | 47 (53.4) |  |
| INR | <0.97 | 62 (44.9) | 20 (40.0) | 42 (47.7) | 0.484 |
|  | ≥0.97 | 76 (55.1) | 30 (60.0) | 46 (52.3) |  |
| PT | <11.1 | 62 (44.9) | 20 (40.0) | 42 (47.7) | 0.484 |
|  | ≥11.1 | 76 (55.1) | 30 (60.0) | 46 (52.3) |  |
| APTT | <27.5 | 62 (44.9) | 24 (48.0) | 38 (43.2) | 0.712 |
|  | ≥27.5 | 76 (55.1) | 26 (52.0) | 50 (56.8) |  |
| TT | <17.2 | 66 (47.8) | 22 (44.0) | 44 (50.0) | 0.616 |
|  | ≥17.2 | 72 (52.2) | 28 (56.0) | 44 (50.0) |  |
| Blood type | A | 31 (22.5) | 10 (20.0) | 21 (23.9) | 0.607 |
|  | B | 51 (37.0) | 16 (32.0) | 35 (39.8) |  |
|  | O | 45 (32.6) | 19 (38.0) | 26 (29.5) |  |
|  | AB | 11 (8.0) | 5 (10.0) | 6 (6.8) |  |
| White blood cell | <5.45 | 63 (45.7) | 20 (40.0) | 43 (48.9) | 0.408 |
|  | ≥5.45 | 75 (54.3) | 30 (60.0) | 45 (51.1) |  |
| Red blood cell | <4.2 | 72 (52.2) | 22 (44.0) | 50 (56.8) | 0.203 |
|  | ≥4.2 | 66 (47.8) | 28 (56.0) | 38 (43.2) |  |
| Hemoglobin | <127 | 75 (54.3) | 24 (48.0) | 51 (58.0) | 0.342 |
|  | ≥127 | 63 (45.7) | 26 (52.0) | 37 (42.0) |  |
| Neutrophil | <3.23 | 67 (48.6) | 22 (44.0) | 45 (51.1) | 0.529 |
|  | ≥3.23 | 71 (51.4) | 28 (56.0) | 43 (48.9) |  |
| Lymphocyte | <1.70 | 62 (44.9) | 22 (44.0) | 40 (45.5) | 1.000 |
|  | ≥1.70 | 76 (55.1) | 28 (56.0) | 48 (54.5) |  |
| Monocyte | <0.35 | 55 (39.9) | 21 (42.0) | 34 (38.6) | 0.836 |
|  | ≥0.35 | 83 (60.1) | 29 (58.0) | 54 (61.4) |  |
| Eosinophil | <0.07 | 72 (52.2) | 24 (48.0) | 48 (54.5) | 0.574 |
|  | ≥0.07 | 66 (47.8) | 26 (52.0) | 40 (45.5) |  |
| Basophil | <0.02 | 71 (51.4) | 22 (44.0) | 49 (55.7) | 0.253 |
|  | ≥0.02 | 67 (48.6) | 28 (56.0) | 39 (44.3) |  |
| Platelet | <233 | 60 (43.5) | 18 (36.0) | 42 (47.7) | 0.247 |
|  | ≥233 | 78 (56.5) | 32 (64.0) | 46 (52.3) |  |
| Primary tumor site | Upper outer quadrant | 80 (58.0) | 30 (60.0) | 50 (56.8) | 0.133 |
|  | Lower outer quadrant | 13 (9.4) | 3 (6.0) | 10 (11.4) |  |
|  | Lower inner quadrant | 12 (8.7) | 8 (16.0) | 4 (4.5) |  |
|  | Upper inner quadrant | 17 (12.3) | 5 (10.0) | 12 (13.6) |  |
|  | Central | 16 (11.6) | 4 (8.0) | 12 (13.6) |  |
| US-BIRADS | BIRADS 4 | 86 (62.3) | 33 (66.0) | 53 (60.2) | 0.754 |
|  | BIRADS 5 | 48 (34.8) | 16 (32.0) | 32 (36.4) |  |
|  | BIRADS 6 | 4 (2.9) | 1 (2.0) | 3 (3.4) |  |
| Operative time | <75 | 66 (47.8) | 20 (40.0) | 46 (52.3) | 0.226 |
|  | ≥75 | 72 (52.2) | 30 (60.0) | 42 (47.7) |  |
| Type of surgery | Mastectomy | 132 (95.7) | 46 (92.0) | 86 (97.7) | 0.249 |
|  | Breast-conserving surgery | 6 (4.3) | 4 (8.0) | 2 (2.3) |  |
| Tumor size | ≤2 | 52 (37.7) | 23 (46.0) | 29 (33.0) | 0.254 |
|  | ＞2 and＜5 | 85 (61.6) | 27 (54.0) | 58 (65.9) |  |
|  | ≥5 | 1 (0.7) | 0 (0.0) | 1 (1.1) |  |
| Histologic grade | I | 4 (2.9) | 3 (6.0) | 1 (1.1) | 0.255 |
|  | II | 80 (58.0) | 31 (62.0) | 49 (55.7) |  |
|  | III | 49 (35.5) | 15 (30.0) | 34 (38.6) |  |
|  | Unknown | 5 (3.6) | 1 (2.0) | 4 (4.5) |  |
| TLN | <16 | 63 (45.7) | 30 (60.0) | 33 (37.5) | 0.018 |
|  | ≥16 | 75 (54.3) | 20 (40.0) | 55 (62.5) |  |
| PLN | <1 | 49 (35.5) | 14 (28.0) | 35 (39.8) | 0.229 |
|  | ≥1 | 89 (64.5) | 36 (72.0) | 53 (60.2) |  |
| TALN | <14 | 67 (48.6) | 33 (66.0) | 34 (38.6) | 0.004 |
|  | ≥14 | 71 (51.4) | 17 (34.0) | 54 (61.4) |  |
| PALN | <1 | 70 (50.7) | 23 (46.0) | 47 (53.4) | 0.509 |
|  | ≥1 | 68 (49.3) | 27 (54.0) | 41 (46.6) |  |
| Molecular subtype | Luminal A | 21 (15.2) | 9 (18.0) | 12 (13.6) | 0.893 |
|  | Luminal B HER2+ | 29 (21.0) | 10 (20.0) | 19 (21.6) |  |
|  | Luminal B HER2- | 32 (23.2) | 13 (26.0) | 19 (21.6) |  |
|  | HER2 enriched | 28 (20.3) | 9 (18.0) | 19 (21.6) |  |
|  | Triple negative | 28 (20.3) | 9 (18.0) | 19 (21.6) |  |
| ER | 0-25% | 62 (44.9) | 21 (42.0) | 41 (46.6) | 0.551 |
|  | 26-50% | 10 (7.2) | 2 (4.0) | 8 (9.1) |  |
|  | 51-75% | 24 (17.4) | 9 (18.0) | 15 (17.0) |  |
|  | 76-100% | 42 (30.4) | 18 (36.0) | 24 (27.3) |  |
| PR | 0-25% | 81 (58.7) | 27 (54.0) | 54 (61.4) | 0.857 |
|  | 26-50% | 18 (13.0) | 7 (14.0) | 11 (12.5) |  |
|  | 51-75% | 14 (10.1) | 6 (12.0) | 8 (9.1) |  |
|  | 76-100% | 25 (18.1) | 10 (20.0) | 15 (17.0) |  |
| HER2 | Negative | 81 (58.7) | 31 (62.0) | 50 (56.8) | 0.679 |
|  | Positive | 57 (41.3) | 19 (38.0) | 38 (43.2) |  |
| Ki67 | 0-25% | 65 (47.1) | 28 (56.0) | 37 (42.0) | 0.375 |
|  | 26-50% | 46 (33.3) | 15 (30.0) | 31 (35.2) |  |
|  | 51-75% | 21 (15.2) | 6 (12.0) | 15 (17.0) |  |
|  | 76-100% | 6 (4.3) | 1 (2.0) | 5 (5.7) |  |
| CK5/6 | Negative | 100 (72.5) | 37 (74.0) | 63 (71.6) | 0.915 |
|  | Positive | 38 (27.5) | 13 (26.0) | 25 (28.4) |  |
| E-cad | Negative | 4 (2.9) | 2 (4.0) | 2 (2.3) | 0.957 |
|  | Positive | 134 (97.1) | 48 (96.0) | 86 (97.7) |  |
| P120 | Negative | 127 (92.0) | 45 (90.0) | 82 (93.2) | 0.737 |
|  | Positive | 11 (8.0) | 5 (10.0) | 6 (6.8) |  |
| P53 | Negative | 76 (55.1) | 35 (70.0) | 41 (46.6) | 0.013 |
|  | Positive | 62 (44.9) | 15 (30.0) | 47 (53.4) |  |
| Blood vessel invasion | No | 134 (97.1) | 49 (98.0) | 85 (96.6) | 1.000 |
|  | Yes | 4 (2.9) | 1 (2.0) | 3 (3.4) |  |
| Lung metastasis | No | 128 (92.8) | 46 (92.0) | 82 (93.2) | 1.000 |
|  | Yes | 10 (7.2) | 4 (8.0) | 6 (6.8) |  |
| Bone metastasis | No | 121 (87.7) | 45 (90.0) | 76 (86.4) | 0.722 |
|  | Yes | 17 (12.3) | 5 (10.0) | 12 (13.6) |  |
| Liver metastasis | No | 128 (92.8) | 46 (92.0) | 82 (93.2) | 1.000 |
|  | Yes | 10 (7.2) | 4 (8.0) | 6 (6.8) |  |
| Mediastinal metastasis | No | 132 (95.7) | 48 (96.0) | 84 (95.5) | 1.000 |
|  | Yes | 6 (4.3) | 2 (4.0) | 4 (4.5) |  |
| Brain metastasis | No | 135 (97.8) | 49 (98.0) | 86 (97.7) | 1.000 |
|  | Yes | 3 (2.2) | 1 (2.0) | 2 (2.3) |  |
| Pleural metastasis | No | 135 (97.8) | 49 (98.0) | 86 (97.7) | 1.000 |
|  | Yes | 3 (2.2) | 1 (2.0) | 2 (2.3) |  |
| Chest wall metastasis | No | 132 (95.7) | 49 (98.0) | 83 (94.3) | 0.558 |
|  | Yes | 6 (4.3) | 1 (2.0) | 5 (5.7) |  |
| Axillary metastasis | No | 69 (50.0) | 22 (44.0) | 47 (53.4) | 0.376 |
|  | Yes | 69 (50.0) | 28 (56.0) | 41 (46.6) |  |
| Clavicle metastasis | No | 119 (86.2) | 44 (88.0) | 75 (85.2) | 0.844 |
|  | Yes | 19 (13.8) | 6 (12.0) | 13 (14.8) |  |
| Chemotherapy | No | 5 (3.6) | 1 (2.0) | 4 (4.5) | 0.768 |
|  | Yes | 133 (96.4) | 49 (98.0) | 84 (95.5) |  |
| Radiotherapy | No | 113 (81.9) | 40 (80.0) | 73 (83.0) | 0.839 |
|  | Yes | 25 (18.1) | 10 (20.0) | 15 (17.0) |  |
| Endocrine therapy | No | 66 (47.8) | 20 (40.0) | 46 (52.3) | 0.226 |
|  | Yes | 72 (52.2) | 30 (60.0) | 42 (47.7) |  |
| Targeted therapy | No | 120 (87.0) | 42 (84.0) | 78 (88.6) | 0.607 |
|  | Yes | 18 (13.0) | 8 (16.0) | 10 (11.4) |  |

**Table S6** Demographic and clinicopathologic characteristics of breast cancer patients with stage III

|  | level | Overall | Low TOX | High TOX | p |
| --- | --- | --- | --- | --- | --- |
| n |  | 90 | 43 | 47 |  |
| Age | <51 | 41 (45.6) | 18 (41.9) | 23 (48.9) | 0.645 |
|  | ≥51 | 49 (54.4) | 25 (58.1) | 24 (51.1) |  |
| Weight | <62 | 41 (45.6) | 21 (48.8) | 20 (42.6) | 0.699 |
|  | ≥62 | 49 (54.4) | 22 (51.2) | 27 (57.4) |  |
| Height | <1.60 | 33 (36.7) | 15 (34.9) | 18 (38.3) | 0.907 |
|  | ≥1.60 | 57 (63.3) | 28 (65.1) | 29 (61.7) |  |
| BMI | <23.8 | 44 (48.9) | 21 (48.8) | 23 (48.9) | 1.000 |
|  | ≥23.8 | 46 (51.1) | 22 (51.2) | 24 (51.1) |  |
| Family history | No | 72 (80.0) | 31 (72.1) | 41 (87.2) | 0.126 |
|  | Yes | 18 (20.0) | 12 (27.9) | 6 (12.8) |  |
| Basic disease | No | 71 (78.9) | 35 (81.4) | 36 (76.6) | 0.765 |
|  | Yes | 19 (21.1) | 8 (18.6) | 11 (23.4) |  |
| Hypertension | No | 80 (88.9) | 39 (90.7) | 41 (87.2) | 0.852 |
|  | Yes | 10 (11.1) | 4 (9.3) | 6 (12.8) |  |
| Diabetes mellitus | No | 84 (93.3) | 41 (95.3) | 43 (91.5) | 0.756 |
|  | Yes | 6 (6.7) | 2 (4.7) | 4 (8.5) |  |
| Coronary heart disease | No | 86 (95.6) | 41 (95.3) | 45 (95.7) | 1.000 |
|  | Yes | 4 (4.4) | 2 (4.7) | 2 (4.3) |  |
| Menarche age | <15 | 33 (36.7) | 17 (39.5) | 16 (34.0) | 0.748 |
|  | ≥15 | 57 (63.3) | 26 (60.5) | 31 (66.0) |  |
| Menopause | No | 38 (42.2) | 16 (37.2) | 22 (46.8) | 0.479 |
|  | Yes | 52 (57.8) | 27 (62.8) | 25 (53.2) |  |
| ALT | <21 | 38 (42.2) | 15 (34.9) | 23 (48.9) | 0.257 |
|  | ≥21 | 52 (57.8) | 28 (65.1) | 24 (51.1) |  |
| AST | <23 | 39 (43.3) | 16 (37.2) | 23 (48.9) | 0.364 |
|  | ≥23 | 51 (56.7) | 27 (62.8) | 24 (51.1) |  |
| AST/ALT | <1.1 | 47 (52.2) | 24 (55.8) | 23 (48.9) | 0.659 |
|  | ≥1.1 | 43 (47.8) | 19 (44.2) | 24 (51.1) |  |
| LDH | <170 | 41 (45.6) | 21 (48.8) | 20 (42.6) | 0.699 |
|  | ≥170 | 49 (54.4) | 22 (51.2) | 27 (57.4) |  |
| GGT | <14 | 41 (45.6) | 20 (46.5) | 21 (44.7) | 1.000 |
|  | ≥14 | 49 (54.4) | 23 (53.5) | 26 (55.3) |  |
| ALP | <70 | 39 (43.3) | 18 (41.9) | 21 (44.7) | 0.955 |
|  | ≥70 | 51 (56.7) | 25 (58.1) | 26 (55.3) |  |
| GLU | <5.1 | 47 (52.2) | 23 (53.5) | 24 (51.1) | 0.985 |
|  | ≥5.1 | 43 (47.8) | 20 (46.5) | 23 (48.9) |  |
| ALB | <45 | 41 (45.6) | 22 (51.2) | 19 (40.4) | 0.418 |
|  | ≥45 | 49 (54.4) | 21 (48.8) | 28 (59.6) |  |
| BUN | <4.9 | 41 (45.6) | 17 (39.5) | 24 (51.1) | 0.376 |
|  | ≥4.9 | 49 (54.4) | 26 (60.5) | 23 (48.9) |  |
| UR/CR | <0.078 | 44 (48.9) | 19 (44.2) | 25 (53.2) | 0.520 |
|  | ≥0.078 | 46 (51.1) | 24 (55.8) | 22 (46.8) |  |
| CRE | <63 | 43 (47.8) | 20 (46.5) | 23 (48.9) | 0.985 |
|  | ≥63 | 47 (52.2) | 23 (53.5) | 24 (51.1) |  |
| URIC | <253 | 34 (37.8) | 19 (44.2) | 15 (31.9) | 0.326 |
|  | ≥253 | 56 (62.2) | 24 (55.8) | 32 (68.1) |  |
| TBIL | <12.45 | 35 (38.9) | 14 (32.6) | 21 (44.7) | 0.336 |
|  | ≥12.45 | 55 (61.1) | 29 (67.4) | 26 (55.3) |  |
| DBIL | <3.9 | 33 (36.7) | 14 (32.6) | 19 (40.4) | 0.579 |
|  | ≥3.9 | 57 (63.3) | 29 (67.4) | 28 (59.6) |  |
| IBIL | <8.29 | 38 (42.2) | 15 (34.9) | 23 (48.9) | 0.257 |
|  | ≥8.29 | 52 (57.8) | 28 (65.1) | 24 (51.1) |  |
| TP | <74 | 32 (35.6) | 17 (39.5) | 15 (31.9) | 0.593 |
|  | ≥74 | 58 (64.4) | 26 (60.5) | 32 (68.1) |  |
| G | <29 | 31 (34.4) | 17 (39.5) | 14 (29.8) | 0.453 |
|  | ≥29 | 59 (65.6) | 26 (60.5) | 33 (70.2) |  |
| A/G | <1.5 | 37 (41.1) | 17 (39.5) | 20 (42.6) | 0.939 |
|  | ≥1.5 | 53 (58.9) | 26 (60.5) | 27 (57.4) |  |
| PAB | <267 | 46 (51.1) | 19 (44.2) | 27 (57.4) | 0.296 |
|  | ≥267 | 44 (48.9) | 24 (55.8) | 20 (42.6) |  |
| CO2 | <28.5 | 38 (42.2) | 15 (34.9) | 23 (48.9) | 0.257 |
|  | ≥28.5 | 52 (57.8) | 28 (65.1) | 24 (51.1) |  |
| CA153 | <9.82 | 30 (33.3) | 14 (32.6) | 16 (34.0) | 1.000 |
|  | ≥9.82 | 60 (66.7) | 29 (67.4) | 31 (66.0) |  |
| CEA | <1.49 | 32 (35.6) | 13 (30.2) | 19 (40.4) | 0.430 |
|  | ≥1.49 | 58 (64.4) | 30 (69.8) | 28 (59.6) |  |
| D-D | <0.25 | 40 (44.4) | 24 (55.8) | 16 (34.0) | 0.062 |
|  | ≥0.25 | 50 (55.6) | 19 (44.2) | 31 (66.0) |  |
| FBG | <2.6 | 45 (50.0) | 26 (60.5) | 19 (40.4) | 0.091 |
|  | ≥2.6 | 45 (50.0) | 17 (39.5) | 28 (59.6) |  |
| INR | <0.97 | 39 (43.3) | 23 (53.5) | 16 (34.0) | 0.100 |
|  | ≥0.97 | 51 (56.7) | 20 (46.5) | 31 (66.0) |  |
| PT | <11.1 | 38 (42.2) | 23 (53.5) | 15 (31.9) | 0.063 |
|  | ≥11.1 | 52 (57.8) | 20 (46.5) | 32 (68.1) |  |
| APTT | <27.5 | 50 (55.6) | 22 (51.2) | 28 (59.6) | 0.555 |
|  | ≥27.5 | 40 (44.4) | 21 (48.8) | 19 (40.4) |  |
| TT | <17.2 | 42 (46.7) | 17 (39.5) | 25 (53.2) | 0.278 |
|  | ≥17.2 | 48 (53.3) | 26 (60.5) | 22 (46.8) |  |
| Blood type | A | 21 (23.3) | 10 (23.3) | 11 (23.4) | 0.988 |
|  | B | 35 (38.9) | 16 (37.2) | 19 (40.4) |  |
|  | O | 22 (24.4) | 11 (25.6) | 11 (23.4) |  |
|  | AB | 12 (13.3) | 6 (14.0) | 6 (12.8) |  |
| White blood cell | <5.45 | 43 (47.8) | 22 (51.2) | 21 (44.7) | 0.686 |
|  | ≥5.45 | 47 (52.2) | 21 (48.8) | 26 (55.3) |  |
| Red blood cell | <4.2 | 39 (43.3) | 17 (39.5) | 22 (46.8) | 0.629 |
|  | ≥4.2 | 51 (56.7) | 26 (60.5) | 25 (53.2) |  |
| Hemoglobin | <127 | 35 (38.9) | 14 (32.6) | 21 (44.7) | 0.336 |
|  | ≥127 | 55 (61.1) | 29 (67.4) | 26 (55.3) |  |
| Neutrophil | <3.23 | 41 (45.6) | 24 (55.8) | 17 (36.2) | 0.097 |
|  | ≥3.23 | 49 (54.4) | 19 (44.2) | 30 (63.8) |  |
| Lymphocyte | <1.70 | 50 (55.6) | 23 (53.5) | 27 (57.4) | 0.869 |
|  | ≥1.70 | 40 (44.4) | 20 (46.5) | 20 (42.6) |  |
| Monocyte | <0.35 | 49 (54.4) | 23 (53.5) | 26 (55.3) | 1.000 |
|  | ≥0.35 | 41 (45.6) | 20 (46.5) | 21 (44.7) |  |
| Eosinophil | <0.07 | 42 (46.7) | 22 (51.2) | 20 (42.6) | 0.544 |
|  | ≥0.07 | 48 (53.3) | 21 (48.8) | 27 (57.4) |  |
| Basophil | <0.02 | 36 (40.0) | 17 (39.5) | 19 (40.4) | 1.000 |
|  | ≥0.02 | 54 (60.0) | 26 (60.5) | 28 (59.6) |  |
| Platelet | <233 | 53 (58.9) | 24 (55.8) | 29 (61.7) | 0.724 |
|  | ≥233 | 37 (41.1) | 19 (44.2) | 18 (38.3) |  |
| Primary tumor site | Upper outer quadrant | 53 (58.9) | 25 (58.1) | 28 (59.6) | 0.948 |
|  | Lower outer quadrant | 10 (11.1) | 4 (9.3) | 6 (12.8) |  |
|  | Lower inner quadrant | 3 (3.3) | 2 (4.7) | 1 (2.1) |  |
|  | Upper inner quadrant | 10 (11.1) | 5 (11.6) | 5 (10.6) |  |
|  | Central | 14 (15.6) | 7 (16.3) | 7 (14.9) |  |
| US BIRADS | BIRADS 4 | 27 (30.0) | 12 (27.9) | 15 (31.9) | 0.543 |
|  | BIRADS 5 | 62 (68.9) | 30 (69.8) | 32 (68.1) |  |
|  | BIRADS 6 | 1 (1.1) | 1 (2.3) | 0 (0.0) |  |
| Operative time | <75 | 35 (38.9) | 13 (30.2) | 22 (46.8) | 0.163 |
|  | ≥75 | 55 (61.1) | 30 (69.8) | 25 (53.2) |  |
| Tumor size | ≤2 | 26 (28.9) | 12 (27.9) | 14 (29.8) | 0.949 |
|  | ＞2 and＜5 | 55 (61.1) | 27 (62.8) | 28 (59.6) |  |
|  | ≥5 | 9 (10.0) | 4 (9.3) | 5 (10.6) |  |
| Histologic grade | I | 47 (52.2) | 23 (53.5) | 24 (51.1) | 0.197 |
|  | II | 35 (38.9) | 14 (32.6) | 21 (44.7) |  |
|  | III | 8 (8.9) | 6 (14.0) | 2 (4.3) |  |
|  | Unknown |  |  |  |  |
| Molecular subtype | Luminal A | 13 (14.4) | 7 (16.3) | 6 (12.8) | 0.962 |
|  | Luminal B HER2+ | 16 (17.8) | 8 (18.6) | 8 (17.0) |  |
|  | Luminal B HER2- | 24 (26.7) | 10 (23.3) | 14 (29.8) |  |
|  | HER2 enriched | 23 (25.6) | 11 (25.6) | 12 (25.5) |  |
|  | Triple negative | 14 (15.6) | 7 (16.3) | 7 (14.9) |  |
| ER | 0-25% | 44 (48.9) | 24 (55.8) | 20 (42.6) | 0.343 |
|  | 26-50% | 7 (7.8) | 4 (9.3) | 3 (6.4) |  |
|  | 51-75% | 12 (13.3) | 6 (14.0) | 6 (12.8) |  |
|  | 76-100% | 27 (30.0) | 9 (20.9) | 18 (38.3) |  |
| PR | 0-25% | 60 (66.7) | 32 (74.4) | 28 (59.6) | 0.051 |
|  | 26-50% | 6 (6.7) | 0 (0.0) | 6 (12.8) |  |
|  | 51-75% | 12 (13.3) | 4 (9.3) | 8 (17.0) |  |
|  | 76-100% | 12 (13.3) | 7 (16.3) | 5 (10.6) |  |
| HER2 | Negative | 51 (56.7) | 24 (55.8) | 27 (57.4) | 1.000 |
|  | Positive | 39 (43.3) | 19 (44.2) | 20 (42.6) |  |
| Ki67 | 0-25% | 37 (41.1) | 21 (48.8) | 16 (34.0) | 0.550 |
|  | 26-50% | 35 (38.9) | 15 (34.9) | 20 (42.6) |  |
|  | 51-75% | 13 (14.4) | 5 (11.6) | 8 (17.0) |  |
|  | 76-100% | 5 (5.6) | 2 (4.7) | 3 (6.4) |  |
| CK5/6 | Negative | 63 (70.0) | 30 (69.8) | 33 (70.2) | 1.000 |
|  | Positive | 27 (30.0) | 13 (30.2) | 14 (29.8) |  |
| E-cad | Negative | 3 (3.3) | 2 (4.7) | 1 (2.1) | 0.938 |
|  | Positive | 87 (96.7) | 41 (95.3) | 46 (97.9) |  |
| P120 | Negative | 83 (92.2) | 38 (88.4) | 45 (95.7) | 0.363 |
|  | Positive | 7 (7.8) | 5 (11.6) | 2 (4.3) |  |
| P53 | Negative | 47 (52.2) | 23 (53.5) | 24 (51.1) | 0.985 |
|  | Positive | 43 (47.8) | 20 (46.5) | 23 (48.9) |  |
| Blood vessel invasion | No | 75 (83.3) | 36 (83.7) | 39 (83.0) | 1.000 |
|  | Yes | 15 (16.7) | 7 (16.3) | 8 (17.0) |  |
| Lung metastasis | No | 74 (82.2) | 35 (81.4) | 39 (83.0) | 1.000 |
|  | Yes | 16 (17.8) | 8 (18.6) | 8 (17.0) |  |
| Bone metastasis | No | 70 (77.8) | 30 (69.8) | 40 (85.1) | 0.135 |
|  | Yes | 20 (22.2) | 13 (30.2) | 7 (14.9) |  |
| Liver metastasis | No | 75 (83.3) | 33 (76.7) | 42 (89.4) | 0.186 |
|  | Yes | 15 (16.7) | 10 (23.3) | 5 (10.6) |  |
| Mediastinal metastasis | No | 85 (94.4) | 39 (90.7) | 46 (97.9) | 0.306 |
|  | Yes | 5 (5.6) | 4 (9.3) | 1 (2.1) |  |
| Brain metastasis | No | 80 (88.9) | 36 (83.7) | 44 (93.6) | 0.247 |
|  | Yes | 10 (11.1) | 7 (16.3) | 3 (6.4) |  |
| Pleural metastasis | No | 84 (93.3) | 39 (90.7) | 45 (95.7) | 0.592 |
|  | Yes | 6 (6.7) | 4 (9.3) | 2 (4.3) |  |
| Chest wall metastasis | No | 85 (94.4) | 40 (93.0) | 45 (95.7) | 0.918 |
|  | Yes | 5 (5.6) | 3 (7.0) | 2 (4.3) |  |
| Axillary metastasis | No | 5 (5.6) | 3 (7.0) | 2 (4.3) | 0.918 |
|  | Yes | 85 (94.4) | 40 (93.0) | 45 (95.7) |  |
| Clavicle metastasis | No | 68 (75.6) | 33 (76.7) | 35 (74.5) | 0.996 |
|  | Yes | 22 (24.4) | 10 (23.3) | 12 (25.5) |  |
| Chemotherapy | No | 3 (3.3) | 2 (4.7) | 1 (2.1) | 0.938 |
|  | Yes | 87 (96.7) | 41 (95.3) | 46 (97.9) |  |
| Radiotherapy | No | 34 (37.8) | 16 (37.2) | 18 (38.3) | 1.000 |
|  | Yes | 56 (62.2) | 27 (62.8) | 29 (61.7) |  |
| Endocrine therapy | No | 42 (46.7) | 20 (46.5) | 22 (46.8) | 1.000 |
|  | Yes | 48 (53.3) | 23 (53.5) | 25 (53.2) |  |
| Targeted therapy | No | 81 (90.0) | 39 (90.7) | 42 (89.4) | 1.000 |
|  | Yes | 9 (10.0) | 4 (9.3) | 5 (10.6) |  |

**Figure S1** the representative figures for the expression of TOX.


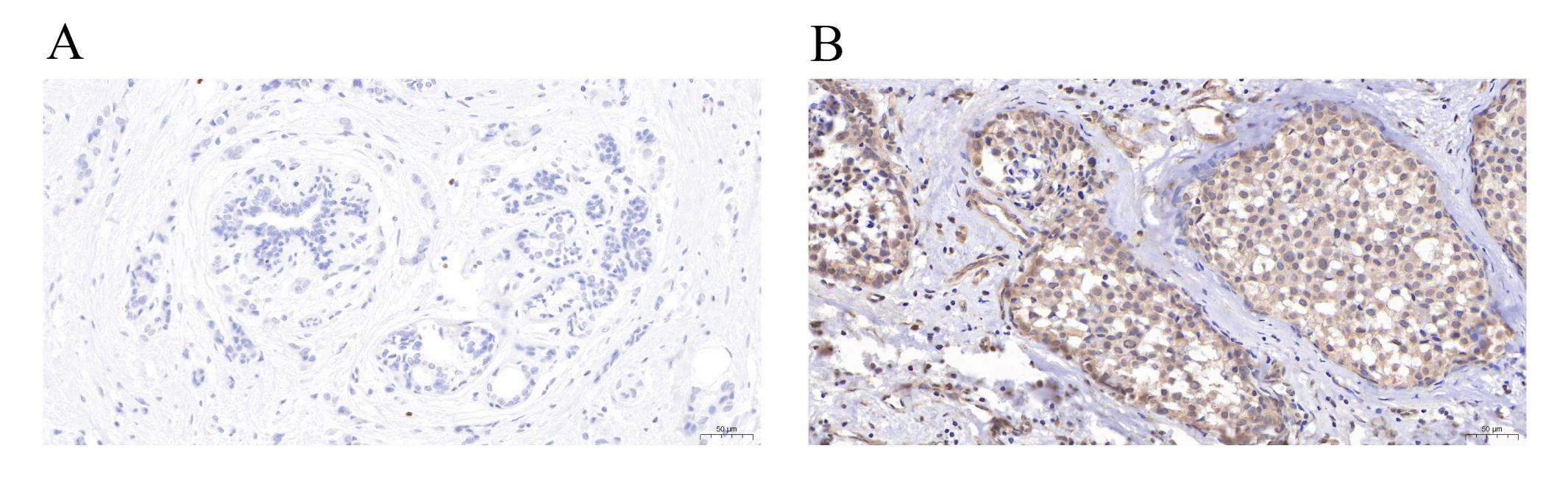


**Figure S2** Survival curve of TOX expression level and prognosis in patients with breast cancer for (A) recurrence free survival and (B) overall survival in Kaplan-Meier Plotter database.


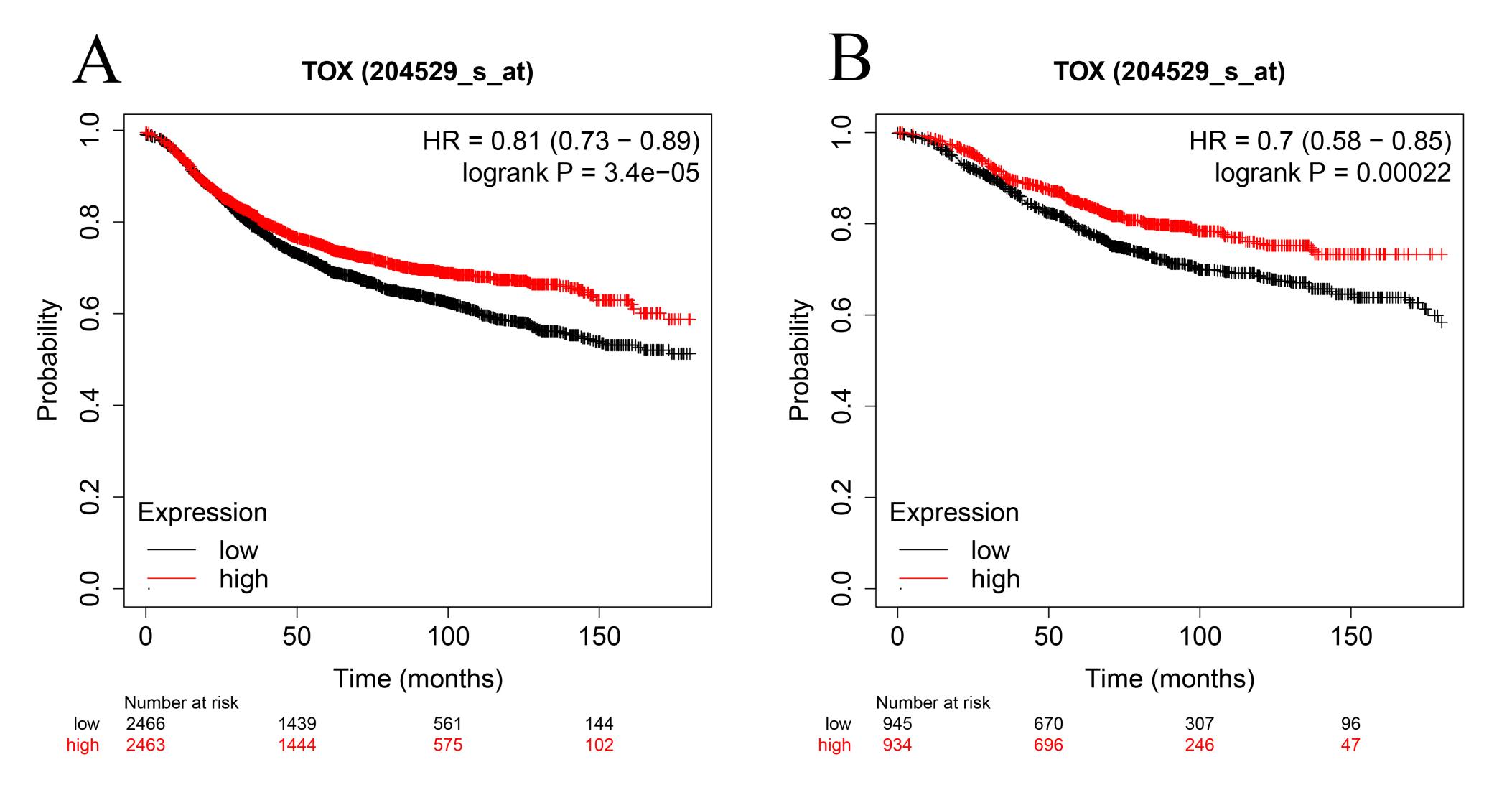


**Figure S3** Survival curve of TOX expression level and prognosis in patients with breast cancer received chemotherapy for (A) disease free survival and (B) overall survival.


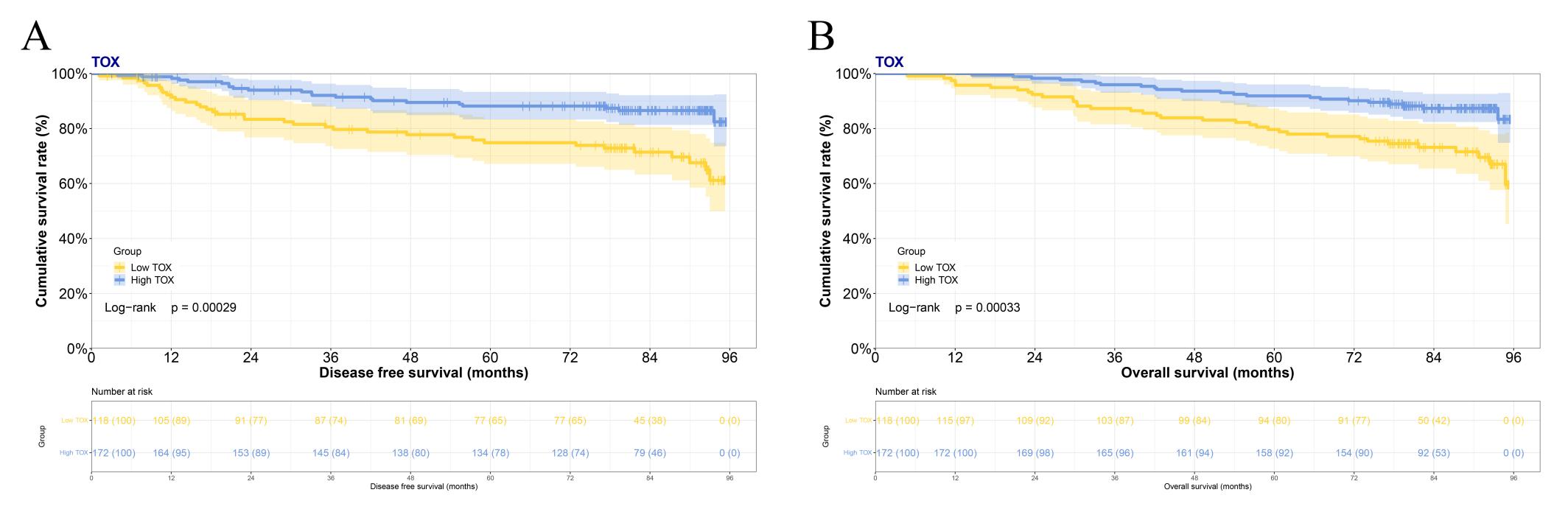


**Figure S4** Survival curve of TOX expression level and prognosis in patients with breast cancer received endocrine therapy for (A) disease free survival and (B) overall survival.


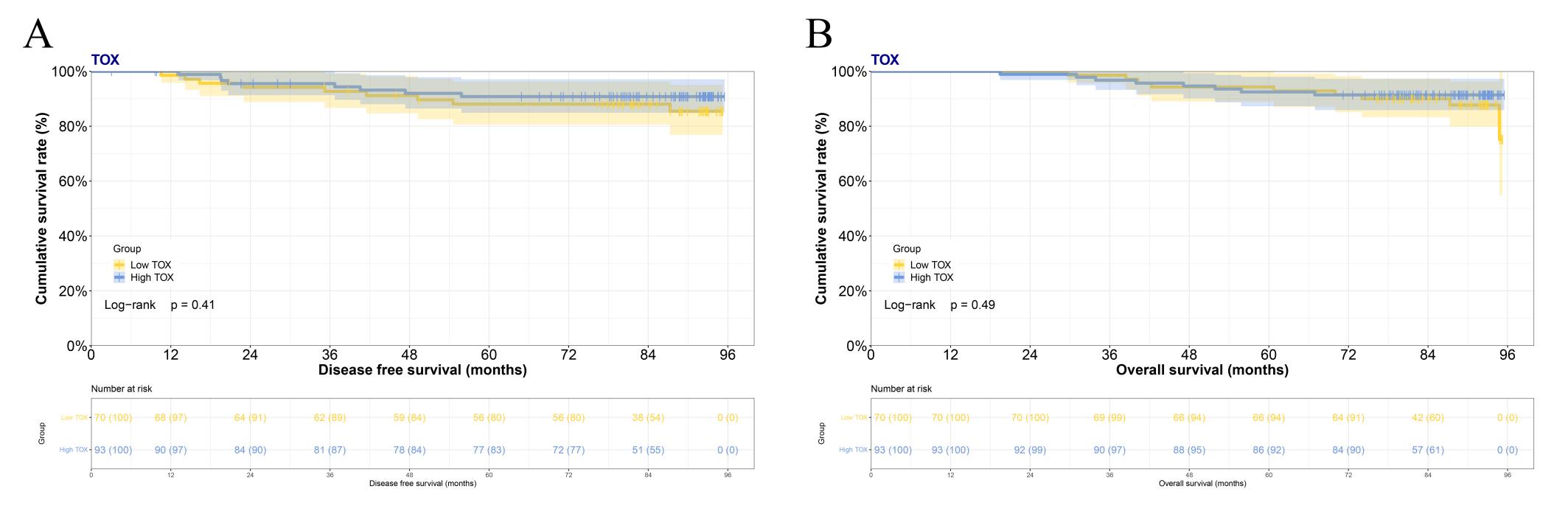


**Figure S5** Survival curve of TOX expression level and prognosis in patients with breast cancer received radiotherapy for (A) disease free survival and (B) overall survival.


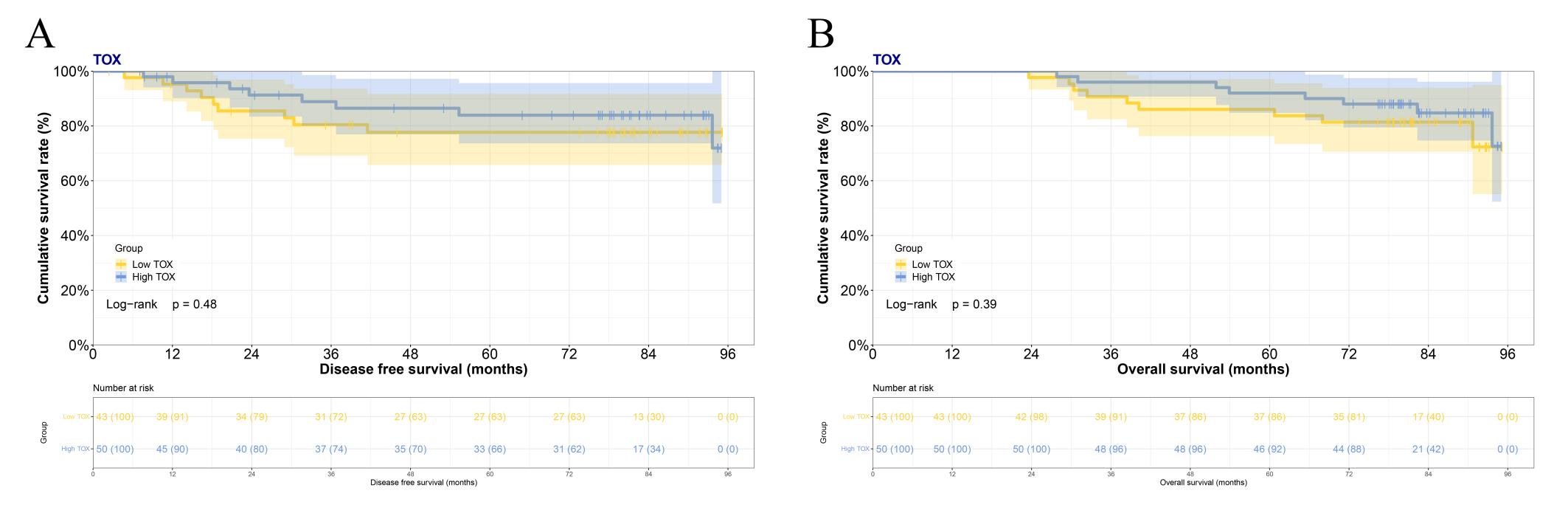


**Figure S6** Survival curve of TOX expression level and prognosis in breast cancer patients with stage I for (A) disease free survival and (B) overall survival.


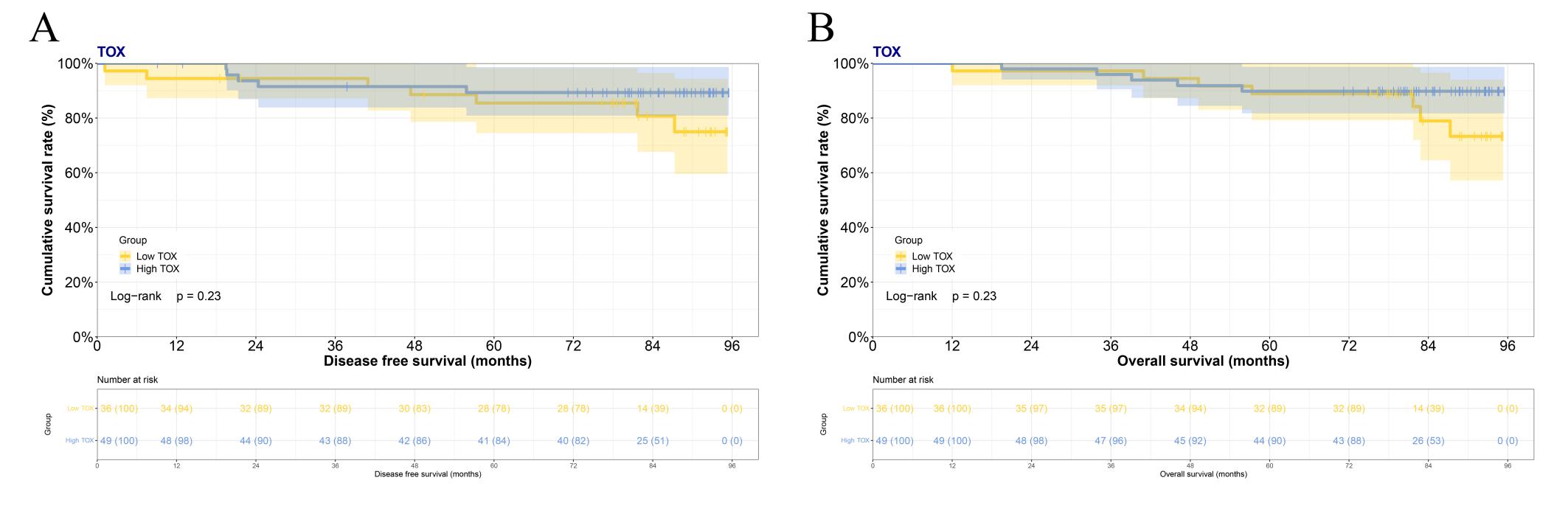


**Figure S7** Survival curve of TOX expression level and prognosis in breast cancer patients with stage II for (A) disease free survival and (B) overall survival.


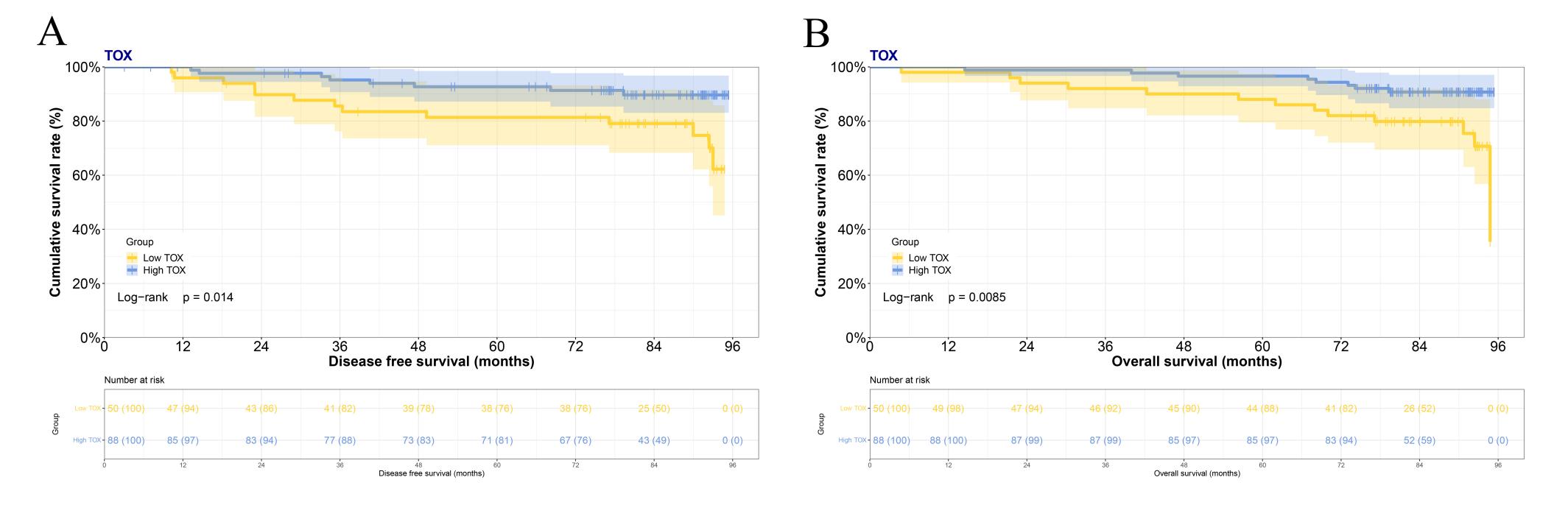


**Figure S8** Survival curve of TOX expression level and prognosis in breast cancer patients with stage III for (A) disease free survival and (B) overall survival.


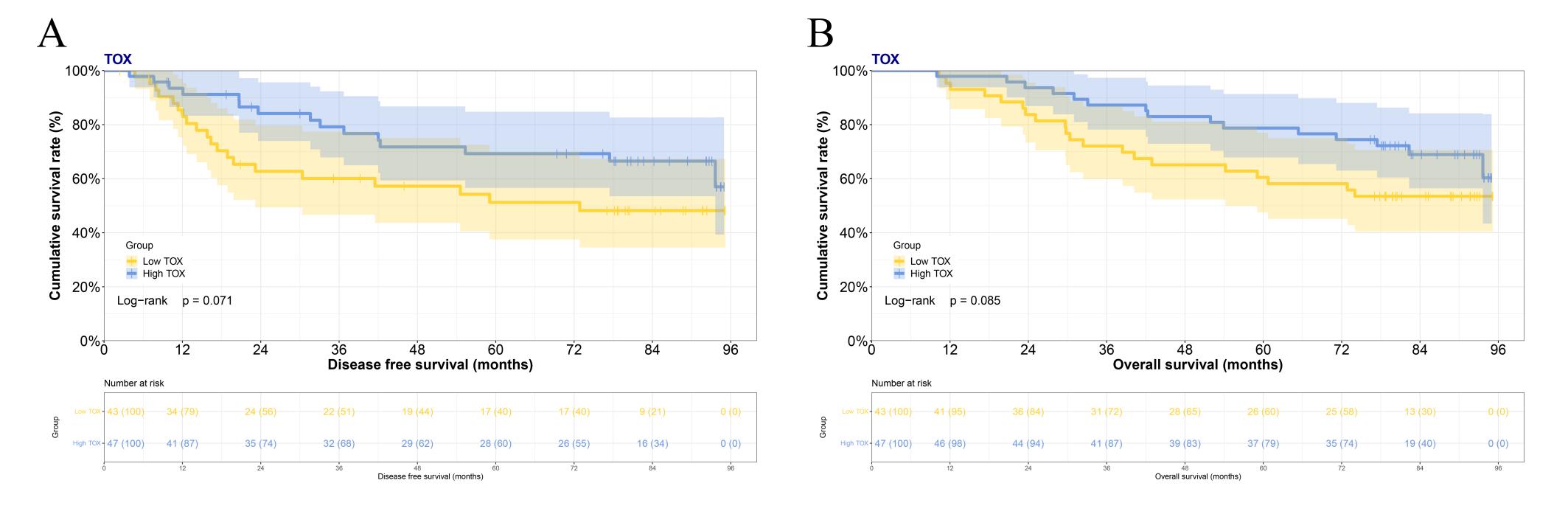

Supplement: Multimedia component 1 [file mmc1.docx]
